# GSK-3β orchestrates the inhibitory innervation of adult-born dentate granule cells in vivo

**DOI:** 10.1007/s00018-023-04874-w

**Published:** 2023-07-23

**Authors:** E. P. Moreno-Jiménez, M. Flor-García, A. Hernández-Vivanco, J. Terreros-Roncal, C. B. Rodríguez-Moreno, N. Toni, P. Méndez, María Llorens-Martín

**Affiliations:** 1 Department of Molecular Neuropathology, Centro de Biología Molecular Severo Ochoa (CBMSO), Spanish Research Council (CSIC), Universidad Autónoma de Madrid (UAM) (Campus de Cantoblanco), c/Nicolás Cabrera 1, 28049 Madrid, Spain; 2 Center for Networked Biomedical Research on Neurodegenerative Diseases (CIBERNED), Madrid, Spain; 3 Department of Molecular Biology, Faculty of Sciences, Universidad Autónoma de Madrid, Madrid, Spain; 4 Cajal Institute, CSIC, Madrid, Spain; 5 Department of Psychiatry, Center for Psychiatric Neurosciences, , Lausanne University Hospital (CHUV) and University of Lausanne, Lausanne, Switzerland

**Keywords:** Adult hippocampal neurogenesis, Gephyrin, Alzheimer´s disease, GSK-3β, Retrovirus, Electrophysiology, Behavior

## Abstract

Adult hippocampal neurogenesis enhances brain plasticity and contributes to the cognitive reserve during aging. Adult hippocampal neurogenesis is impaired in neurological disorders, yet the molecular mechanisms regulating the maturation and synaptic integration of new neurons have not been fully elucidated. GABA is a master regulator of adult and developmental neurogenesis. Here we engineered a novel retrovirus encoding the fusion protein Gephyrin:GFP to longitudinally study the formation and maturation of inhibitory synapses during adult hippocampal neurogenesis in vivo. Our data reveal the early assembly of inhibitory postsynaptic densities at 1 week of cell age. Glycogen synthase kinase 3 Beta (GSK-3β) emerges as a key regulator of inhibitory synapse formation and maturation during adult hippocampal neurogenesis. GSK-3β-overexpressing newborn neurons show an increased number and altered size of Gephyrin^+^ postsynaptic clusters, enhanced miniature inhibitory postsynaptic currents, shorter and distanced axon initial segments, reduced synaptic output at the CA3 and CA2 hippocampal regions, and impaired pattern separation. Moreover, GSK-3β overexpression triggers a depletion of Parvalbumin^+^ interneuron perineuronal nets. These alterations might be relevant in the context of neurological diseases in which the activity of GSK-3β is dysregulated.

**Supplementary Information:**

The online version contains supplementary material available at 10.1007/s00018-023-04874-w.

## Introduction

The hippocampus, a cornerstone in learning and memory, is severely targeted by neurodegenerative diseases, including Alzheimer´s disease (AD). One of the mechanisms contributing to the enhanced synaptic plasticity of this structure is the persistence of adult neurogenesis [1, 2]. As a result of adult hippocampal neurogenesis, new dentate granule cells are continuously incorporated into the hippocampal circuitry throughout life. Adult-born dentate granule cells have special electrophysiological properties that facilitate their involvement in hippocampal-dependent functions such as pattern separation, forgetting, and mood regulation [3–5]. During adult hippocampal neurogenesis, immature neurons go through sequential maturation stages before achieving full synaptic integration [6]. These cells follow a stereotypical integration process. First, they receive tonic γ-Aminobutyric acid (GABA)ergic signals, which are followed by the appearance of GABAergic synapses and, finally, glutamate-mediated synaptic inputs [7]. GABA exerts trophic effects at various stages of dentate granule cell development (reviewed in [8]). However, very immature newborn neurons transiently evade GABAergic perisomatic inhibition, a prominent phenomenon in mature counterparts [9, 10]. This evasion results in a high excitation/inhibition balance, conferring immature dentate granule cells low activation threshold and input specificity [11]. These characteristics turn these cells into a highly active neuronal population immersed within a principal layer (the granule cell layer) characterized by sparse activity [12]. It has been shown that early GABAergic transmission arises from GABA spillover rather than direct synaptic contacts [13], that GABA depolarizes very immature newborn dentate granule cells [14], and that GABAergic synapses made onto newborn neurons require several weeks to mature [15, 16].

The synaptic activity of the dentate gyrus is orchestrated by complex feedforward and feedback synaptic loops in which Parvalbumin^+^ GABAergic interneurons play a major role. Acting as robust gamma frequency oscillators in the adult brain, these cells correspond to fast-spiking basket and axoaxonic cells in the hippocampus [17, 18]. Interneurons provide compartment-selective (e.g., at the soma, axon initial segment, and dendrites) inhibition of the principal cells [18]. In the dentate gyrus, most Parvalbumin^+^ interneurons are surrounded by condensed glycosaminoglycan-rich extracellular matrix structures with a heterogeneous composition yet specific organization named perineuronal nets [19, 20]. Perineuronal nets protect neurons and synaptic connections from damaging environmental stressors and oxidative stress [19, 21], participate in signal transduction and the control of neuronal activity and plasticity [19], regulate water homeostasis [22], act as ion buffers [19], and form physical barriers that protect neurons from Amyloid-β and Tau toxicity [23, 24]. The presence of perineuronal nets is positively related to adult hippocampal neurogenesis [20], whereas these structures are degraded in neurodegenerative diseases, including AD [25–27].

Inhibitory synapses are usually formed onto the cell soma or dendritic shaft of the postsynaptic neuron [28, 29], and they occur at pre-existing crossings with presynaptic axons [30], where new GABAergic boutons containing presynaptic active zone proteins, such as Bassoon, appear [31]. At the postsynaptic level, the scaffold protein Gephyrin forms complexes with neurotransmitter receptors and adaptor proteins. These dense molecular assemblies are essential for the formation of inhibitory postsynaptic density condensates. However, the presence of neurotransmitter receptors is required for the initiation of the inhibitory postsynaptic density formation [32, 33]. In addition to alterations in both excitatory synapses and adult hippocampal neurogenesis, mouse models of neurodegenerative diseases such as AD [34], frontotemporal dementia [35], and epilepsy [36] show altered expression of Gephyrin (reviewed in [37]).

AD is the most common form of age-related dementia. Patients with this condition show a progressive loss of episodic memory, as well as other cognitive and psychiatric comorbidities. The histopathological hallmarks of the disease, namely Amyloid-β senile plaques and hyperphosphorylated Tau neurofibrillary tangles, are accompanied by marked synapse loss and neuronal death. In vitro and in vivo studies point to glycogen synthase kinase 3 Beta (GSK-3β), the main kinase that phosphorylates Tau [38], as a cornerstone in AD pathogenesis. In this regard, an over-activation of GSK-3β induced by Amyloid-β [39–41] is observed in the brains of patients with AD [42]. Accordingly, GSK-3β overexpression in mice mimics pathological events occurring in the brains of patients with AD [43]. Mice overexpressing GSK-3β in hippocampal principal neurons show impaired performance in the Morris water maze [44] and novel object recognition [45] and elevated plus maze [46] tests, Tau hyperphosphorylation, reactive astrogliosis and microgliosis, and exacerbated neuronal death [44, 46, 47]. Some of these effects are paralleled by adult hippocampal impairments [48–50] and excitatory synapsis deficits [34]. However, the extent to which inhibitory synapses are also affected remains to be elucidated. In this regard, GSK-3β—a master regulator of synaptic plasticity at excitatory synapses [51]—also regulates GABAergic transmission in vitro through Ser270 Gephyrin phosphorylation [52]. However, an in vivo role of GSK-3β in the regulation of GABAergic transmission has not been confirmed to date.

To address this question, we used retroviral vectors to selectively label the postsynaptic compartment of inhibitory synapses, the axon initial segment, and the active zone of presynaptic terminals of newborn dentate granule cells. Super-resolution and electron microscopy, whole-cell patch clamp recordings, and the behavioral assessment of adult hippocampal neurogenesis-related functions revealed that GSK-3β overexpression altered both the inhibitory inputs and the synaptic output of these cells. These results show that this kinase regulates the inhibitory innervation and synaptic integration of this cell population. The observed alterations thus might be relevant in the context of neurological disorders in which the activity of this kinase is dysregulated.

## Methods

### Experimental design

**Fig. 1:**
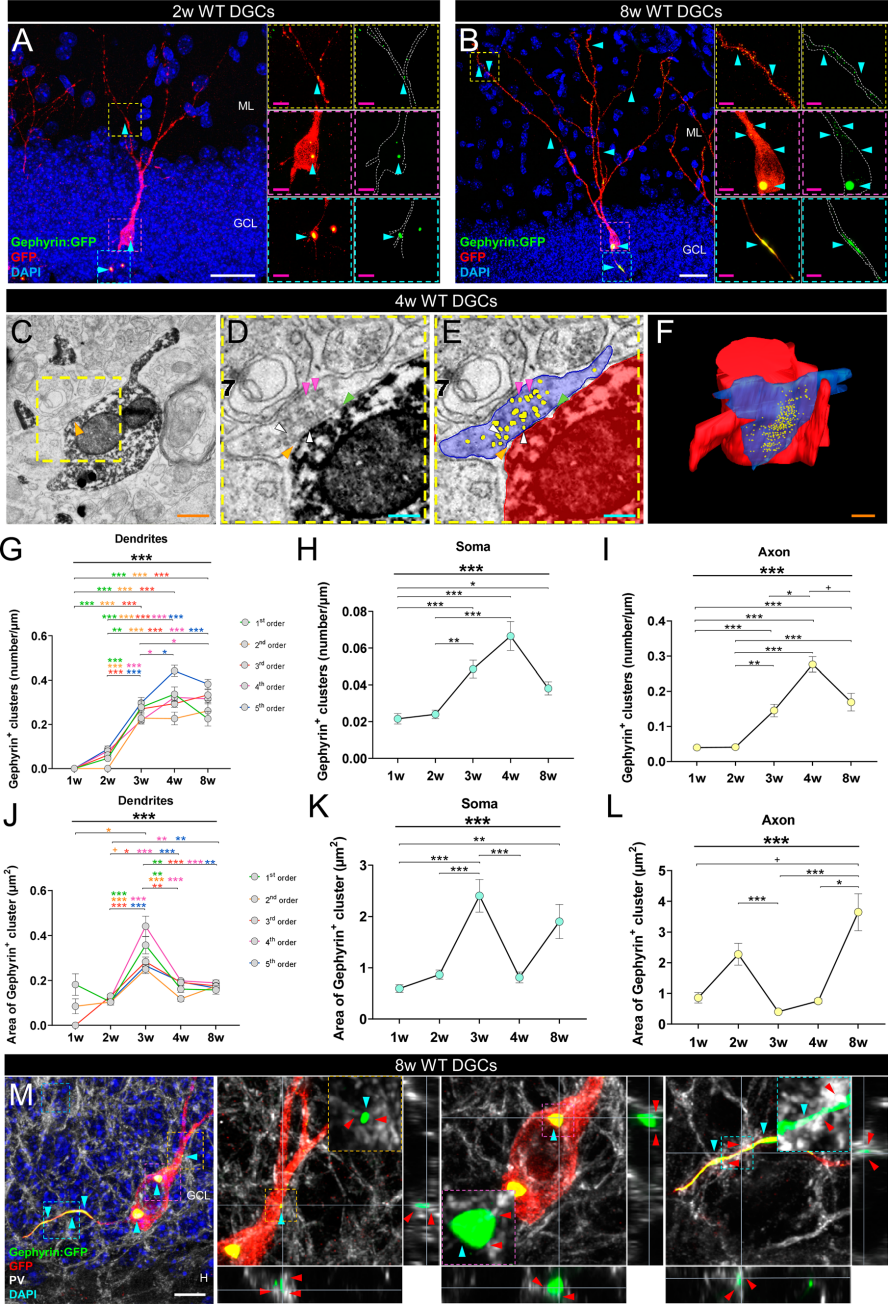
Inhibitory innervation of newborn dentate granule cells (DGCs) of wild-type (WT) mice.** A**, **B** Representative images of 2- (**A**) and 8- (**B**) week-old newborn dentate granule cells transduced with a Gephyrin:GFP-encoding retrovirus. The endogenous fluorescence of the retrovirus (green) enabled visualization of Gephyrin bound to the postsynaptic densities in inhibitory synapses. The use of an anti-GFP antibody (red) enhanced visualization of the intracellular trafficking of the protein and allows visualization of cell morphology [60]. **C**–**F** Representative electron microscopy image (**C–E**) and 3D reconstruction (**F**) of inhibitory synapses made onto 4-week-old newborn dentate granule cells. **G** Density (number/µm) of Gephyrin^+^ clusters in the dendrites of newborn dentate granule cells of distinct ages (1, 2, 3, 4, and 8 weeks post-infection). The density of clusters was evaluated in each dendritic branching order separately. **H–I** Density (number/µm) of Gephyrin^+^ clusters in the soma (**H**) and axon (**I**) of newborn dentate granule cells of distinct ages. **J–L** Area of Gephyrin^+^ clusters in the dendrites (**J**), soma (**K**), and axon (**L**) of newborn dentate granule cells of distinct ages. **M** Representative image of an 8-week-old newborn dentate granule cell transduced with a Gephyrin:GFP-encoding retrovirus, and high-power magnification of Gephyrin^+^ clusters surrounded by Parvalbumin (PV)^+^ terminals together with orthogonal views. In (**A**, **B**, and **M**), Z-projection images are shown. In (**G**–**L**), a nonparametric Kruskal–Wallis test, followed by a Dunn post hoc test, was used. In (**G**, **J**), a minimum of 30 dendritic segments of each branching order (1st, 2nd, 3rd, 4th, and 5th) per cell age, obtained from 4–5 animals per genotype, were analyzed. At least 30 somas (**H**, **K**) and 30 axonal segments (**I**, **L**) per cell age, obtained from 4–5 animals per genotype, were analyzed. Graphs represent mean values ± SEM. ML: Molecular layer. GCL: Granule cell layer. H: Hilus. White scale bar: 25 μm. Magenta scale bar: 5 µm. Orange scale bar: 500 nm. Light blue scale bar: 200 nm. Light blue triangles: Gephyrin^+^ clusters. Orange triangle: inhibitory synapse. White triangles: Readily releasable pool of synaptic vesicles. Magenta triangles: Recycling pool of synaptic vesicles. Green triangles: Synaptic cleft. Red triangles: PV^+^ terminals. ^+^ 0.09 > *p* ≥ 0.05; * 0.05 > *p* ≥ 0.01; ** 0.01 > *p* ≥ 0.001; and *** 0.001 > *p* ≥ 0.0001

**Fig. 2:**
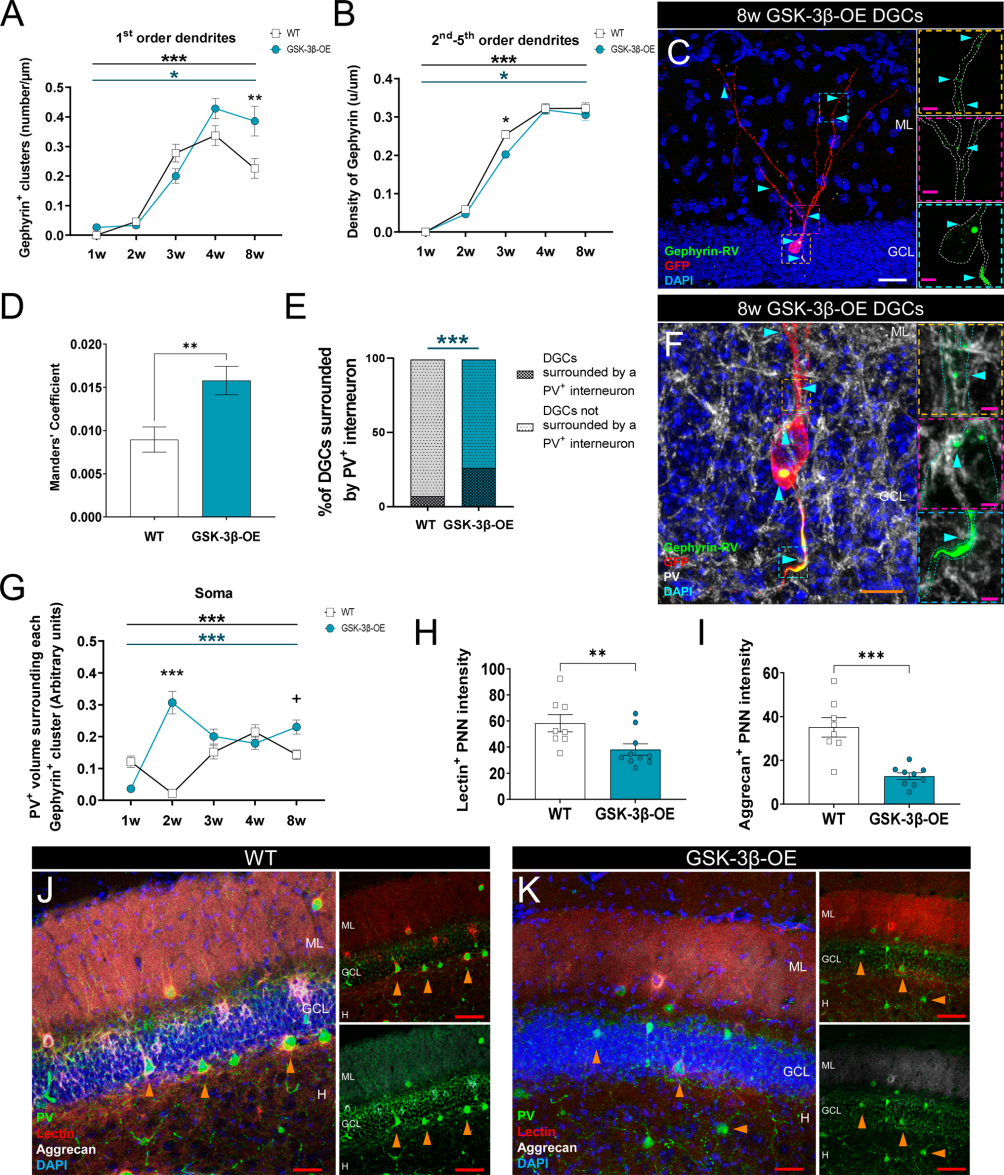
The overexpression of GSK-3β alters the establishment and maturation of inhibitory synaptic contacts made onto newborn dentate granule cells (DGCs). **A**, **B** Density (number/µm) of Gephyrin^+^ clusters in 1st (**A**) and higher branching (**B**) order dendrites of newborn dentate granule cells of distinct ages (1, 2, 3, 4, and 8 weeks post-infection) in wild-type (WT) and GSK-3β-overexpressing (OE) mice. **C** Representative image of an 8-week-old newborn dentate granule cell transduced with a Gephyrin:GFP-encoding retrovirus in a GSK-3β-OE mouse. **D** Colocalization between Bassoon^+^ presynaptic area and Gephyrin^+^ clusters in 4-week-old newborn dentate granule cells of WT and GSK-3β-OE mice. **E** Percentage of GSK-3β-OE 1-week-old newborn dentate granule cells surrounded by Parvalbumin (PV)^+^ interneurons. **F** Representative image of a GSK-3β-OE 8-week-old newborn dentate granule cell transduced with a Gephyrin:GFP-encoding retrovirus, and high-power magnification of axonal Gephyrin^+^ clusters surrounded by PV^+^ terminals. **G** PV^+^ volume surrounding Gephyrin^+^ clusters in the soma of newborn dentate granule cells of distinct ages (1, 2, 3, 4 and 8 weeks post-infection) in WT and GSK-3β-OE mice. **H**, **I** Lectin (**H**) and Aggrecan (**I**) fluorescence intensity in perineuronal nets (PNNs) that surround PV^+^ interneurons in the dentate gyrus. **J**, **K** Representative images of Lectin, Aggrecan, and PV staining in the dentate gyrus of WT (**J**) and GSK-3β-OE (**K**) mice. In (**C**, **F**, **J**, and **K**), Z-projection images are shown. In **A**, **B** and **G**, a two-way ANOVA, followed by a Tukey post hoc test, was used. In (**D**, **H**), a Mann–Whitney *U* test was applied. A Chi-square test was used to analyze the data shown in (**E**). In (**I**), a Student t-test was applied. In **A**, a minimum of 30 dendritic segments of 1st branching order per cell age, obtained from 4–5 animals per genotype, were analyzed. In (**B**), a minimum of 30 dendritic segments of each order (namely 1st, 2nd, 3rd, 4th, and 5th) per cell age, obtained from 4–5 animals per genotype, were analyzed. In **D** and **E**, a minimum of 50 newborn dentate granule cells, obtained from 4–5 animals per genotype, were analyzed. In **G**, at least 30 somatic Gephyrin^+^ clusters per cell age, obtained from 4–5 animals per genotype, were analyzed. In (**H**, **I**), 40–50 stacks of dentate gyrus images, obtained from 8–10 mice per genotype, were analyzed. Graphs represent mean values ± SEM. ML: Molecular layer. GCL: Granule cell layer. H: Hilus. White scale bar: 25 μm. Orange scale bar: 10 μm. Magenta scale bar: 5 µm. Red scale bar: 40 μm. Light blue triangles: Gephyrin^+^ clusters. Orange triangles: PV^+^ interneurons. ^+^ 0.09 > *p* ≥ 0.05; * 0.05 > p ≥ 0.01; ** 0.01 > *p* ≥ 0.001; and *** 0.001 > *p* ≥ 0.0001

**Fig. 3:**
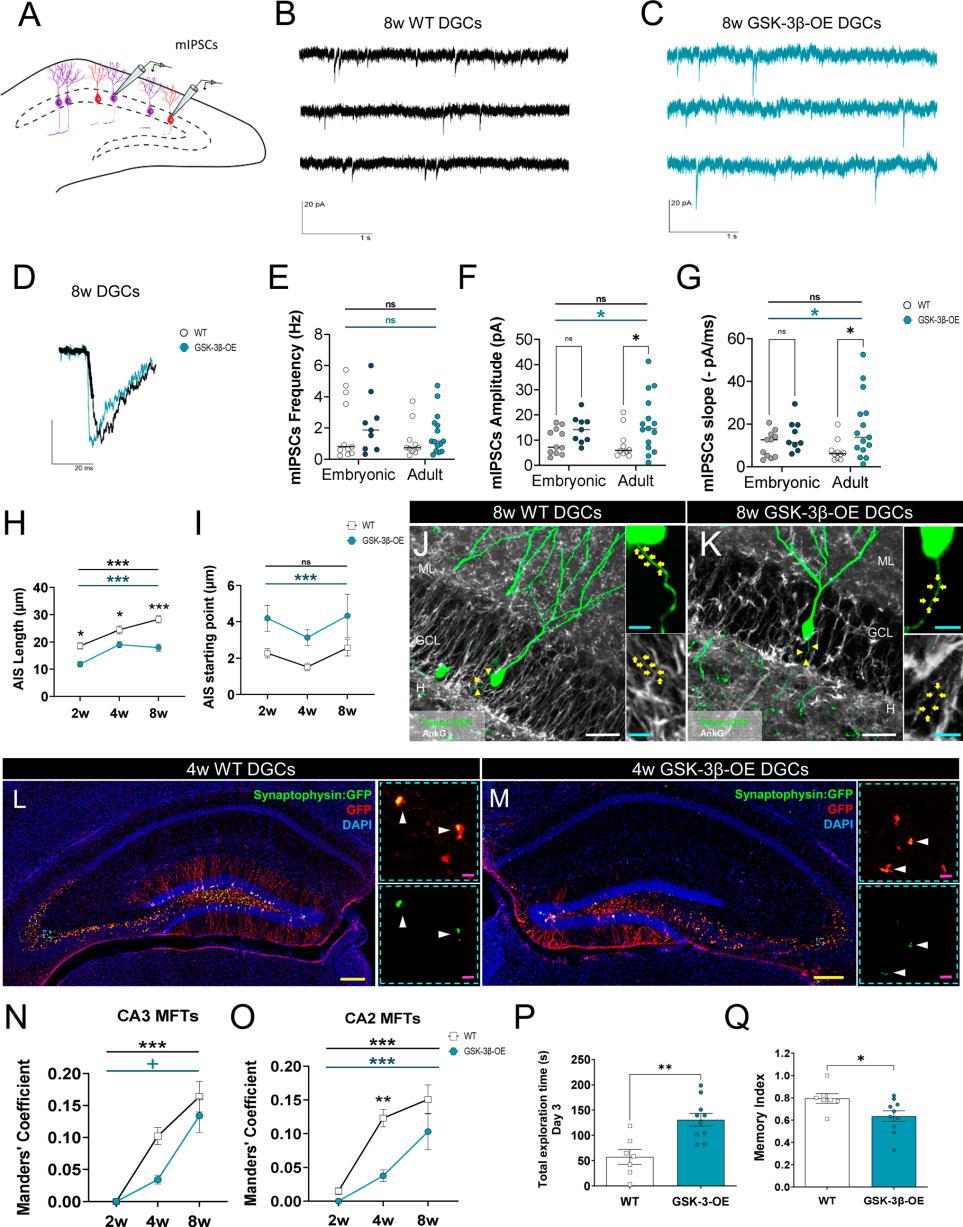
The presynaptic component of inhibitory synapses made onto newborn dentate granule cells (DGCs) is altered in GSK-3β-overexpressing (OE) mice.** A** Schematic diagram illustrating patch-clamp whole-cell recordings and showing 8-week-old newborn dentate granule cells in red and embryonic dentate granule cells in purple. **B**, **C** Representative miniature inhibitory postsynaptic currents (mIPSCs) traces recorded from 8-week-old WT (**B**) and GSK-3β-OE (**C**) dentate granule cells. Vertical deflections represent mIPSCs. **D** Representative normalized traces showing faster rising kinetics in GSK-3β-OE mice. **E–G** Frequency (**E**), amplitude (**F**), and slope (**G**) of mIPSCs in developmentally generated (embryonic) and 8-week-old newborn dentate granule cells (adult-born) in wild-type (WT) (gray and white symbols) and GSK-3β-OE (dark and light blue symbols) mice. **H** Length of the axon initial segment (AIS). **I**: AIS starting point. **J–K** Representative images and high-power magnifications showing the AIS of 8-week-old newborn dentate granule cells transduced with a Venus-encoding retrovirus in WT and GSK-3β-OE mice. **L–M** Representative images and high-power magnifications of 8-week-old newborn dentate granule cells transduced with a Synaptophysin (Syn):GFP-encoding retrovirus in WT and GSK-3β-OE mice. **N–O** Area of CA3 (**N**) and CA2 (**O**) mossy fiber terminals occupied by Syn^+^ clusters (colocalization Mander´s coefficient). **P** Total exploratory time (s) during the second day of the Novel location preference test. **Q**: Memory index on the third day of the Novel location preference test. In (**J–M**), Z-projection images are shown. In (**E–G**), a two-way ANOVA, followed by a Bonferroni post hoc test, was used. A two-way ANOVA, followed by a Tukey post hoc test, was used to analyze the data shown in (**H**, **I**, **N**, and **O**). A Student t-test was applied to analyze the data depicted in (**P**, **Q)**. In (**B–G**), nine developmentally generated (embryonic) and 15 8-week-old dentate granule cells were obtained from eight GSK-3β-OE. These cells were compared to 11 developmentally generated (embryonic) and 10 8-week-old dentate granule cells obtained from four WT mice. In (**H**, **I)**, a minimum of 30 newborn dentate granule cells of each age, obtained from 4–5 animals per genotype, were analyzed. In (**N**, **O)**, a minimum of 10–15 stacks of images per region (CA3 and CA2) and cell age, obtained from 4–5 animals per genotype, were analyzed. In (**P**, **Q)**, eight WT and 10 GSK-3β-OE animals were used. Graphs represent mean values ± SEM. ML: Molecular layer. GCL: Granule cell layer. H: Hilus. White scale bar: 50 μm. Light blue scale bar: 5 μm. Yellow scale bar: 25 μm. Magenta scale bar: 2 µm. Yellow triangles: AIS. White triangles: Syn^+^ clusters. ^+^ 0.09 > *p* ≥ 0.05; * 0.05 > *p* ≥ 0.01; ** 0.01 > *p* ≥ 0.001; and *** 0.001 > *p* ≥ 0.0001

To study the formation and maturation of inhibitory synapses made onto newborn dentate granule cells of wild-type (WT) (Fig. 1 and Supplementary Figs. S1 and S2) and GSK-3β-overexpressing (OE) mice (Fig. 2 and Supplementary Figs. S3–S5), we used a Gephyrin:GFP-encoding retrovirus, which exclusively labels newborn dentate granule cells and allows selective visualization of the postsynaptic densities of their inhibitory synapses. To longitudinally address the morphological properties of Gephyrin^+^ clusters, the retroviruses were stereotaxically injected, and animals were sacrificed at different time points (1, 2, 3, 4, and 8 weeks post-injection) (Supplementary Fig. S1B). The presence of inhibitory synapses made onto newborn dentate granule cells of 4, 8, and 60 weeks of age was further confirmed by electron microscopy (Supplementary Fig. S2). Whole-cell patch-clamp electrophysiological recordings (Fig. 3A–G), axon initial segment (Fig. 3H–K), Synaptophysin(Syn)^+^ area of mossy fiber terminals (Fig. 3L–O), and behavioral tests (Fig. 3P–Q) were analyzed in WT and GSK-3β-OE mice to determine whether the functional output of newborn dentate granule cells was altered in the latter.

### Animals

Five- to six-week-old female C57BL/6J-OlaHsd mice were obtained from Envigo Laboratories. Animals overexpressing GSK-3β under the control of the neuronal promoter of Calcium-Calmodulin kinase II (CamKII) (GSK-3β-OE mice) and WT littermates were generated as previously described [47]. The animals were subjected to a 1- to 2- week habituation period before the experiments began. Three days before stereotaxic injections, a running wheel was included in the cage to enhance stem cell proliferation. The mice were 6–7 weeks old when they received the stereotaxic injections [53–55]. They were housed in a specific pathogen-free colony facility at the *Centro de Biología Molecular “Severo Ochoa”* (CBMSO) in accordance with European Community Guidelines (directive 86/609/EEC) and handled following European and local animal care protocols. Three to five mice were housed per cage. The animal experiments were approved by the CBMSO Ethics Committee (AEEC-CBMSO-23/172) and the National Ethics Committee (PROEX 205/15 and PROEX 185.4/20). For the electron microscopy experiments, 6- to 7-week-old female C57BL/6 J-OlaHsd mice were obtained from Jackson Laboratories. They were subjected to a one-week habituation period in cages with a running wheel to enhance neuronal stem cell proliferation. The animal protocols were approved by the Salk Institutional Animal Care and Use Committee.

### Retroviral stocks

Since the retroviruses used are engineered to be replication-incompetent, only cells dividing at the time of surgery are infected [56]. In the dentate gyrus, these proliferative cells are almost totally restricted to adult-born dentate granule cells [56].

*Gephyrin:GFP- and Gephyrin:mCherry-encoding retroviruses* A Gephyrin:GFP insert was purified from a EGFPC2-Gephyrin P1 plasmid (which was a gift from Shiva Tyagarajan (Addgene plasmid # 68,815#; http://n2t.net/addgene:68815; RRID:Addgene_68815)) and subsequently inserted into a CAG-GFP retroviral vector [56]. The overexpression of Gephyrin:GFP constructs did not influence miniature inhibitory postsynaptic current amplitudes or interevent intervals compared to control cells, thereby indicating that recombinant Gephyrin did not cause measurable overexpression artifacts [52]. To construct a Gephyrin:mCherry-encoding retrovirus, GFP was replaced by mCherry. To this end, GFP was first excised by means of AgeI and HinDIII restriction enzymes. Next, an mCherry-encoding fragment, purified from the Addgene plasmid #55,052# (http://n2t.net/addgene:55052; RRID:Addgene_55052), was subsequently inserted.

*Venus-, PSD95:GFP-, and Synaptophysin:GFP-encoding retroviruses* We used three distinct retroviral stocks encoding for Venus [35, 57–59], PSD95:GFP [60], or Synaptophysin:GFP (Syn:GFP) [61]. The plasmids used to produce the Venus-encoding retroviruses were kindly provided by Profs. Tsien (Howard Hughes Medical Institute Laboratory at the University of California, San Diego, USA), Baum and Schambach (Hannover Medical School, Germany), Miyawaki (RIKEN Brain Science Institute, Saitama, Japan), and Riecken (University Medical Center Hamburg-Eppendorf, Germany), whereas those used to produce the PSD95:GFP and Syn:GFP retroviruses were kindly provided by Prof. Lois (Caltech, Pasadena, USA). We used a Venus-encoding retrovirus to visualize the axon initial segment of newborn dentate granule cells [35]. Syn:GFP-encoding retroviruses were used to visualize the presynaptic active area of newborn dentate granule cell mossy fiber terminals.

The plasmids used to package all the retroviral particles were kindly provided by Prof. Gage (Salk Institute, California, USA). Retroviral stocks were concentrated to working titers of 1 × 10^7^–2 × 10^8^ pfu/ml by ultracentrifugation [56].

*GFP-encoding retroviruses* For the electron microscopy experiments, a retroviral vector based on the Moloney murine leukemia virus (MoMulV) that encodes enhanced GFP was used to visualize adult-born dentate granule cells. Supernatant containing the virus was collected from 293gp/NIT-GFPc11 cells after transfection with pVSVG, as described previously [62]. The plasmids were kindly provided by Prof. Gage. Final viral titers were 5 × 10^8^ pfu/ml.

### Stereotaxic surgery

The mice were anesthetized with isoflourane and placed in a stereotaxic frame. The viruses were injected into the dentate gyrus at the following coordinates (mm) relative to bregma in the anteroposterior, mediolateral, and dorsoventral axes: [– 2.0, ± 1.4, 2.2]. Next, 2 µl of virus was infused at a rate of 0.2 µl/min via a glass micropipette. To avoid a suction effect, pipettes were kept in place at the site of injection for an additional 5 min before being slowly removed. For the electron microscopy experiments, the mice were anesthetized (100 mg ketamine, 10 mg xylazine in 10 ml saline per gram) and infused with 1.5 µl of MoMulV. The virus was injected into the right dentate gyrus at the following coordinates (mm) relative to bregma in the anteroposterior, mediolateral, and dorsoventral axes: [– 2.0, 1.5, 2.0]. Three days after surgery, the mice were placed in standard cages and sacrificed 4, 8, or 60 weeks post-infection. The brains were subsequently processed for electron microscopy as described below.

### Sacrifice

The mice were fully anesthetized by an intraperitoneal injection of pentobarbital (EutaLender, 60 mg/kg). Those used to perform immunohistochemistry (IHC) were transcardially perfused with 0.9% saline followed by 4% paraformaldehyde in 0.1 N phosphate buffer (PB). The PFA fixative solution (pH = 7.4) was freshly prepared immediately before sacrifice by diluting a commercial 16% PFA solution (Electron Microscopy Sciences) in 0.2 N phosphate buffer (PB) and bi-distilled water (1:2:1). The mice used for electron microscopy were transcardially perfused with a solution of PB (pH 7.4) containing 4% PFA and 0.2% glutaraldehyde. The brains were removed and post-fixed either overnight (O/N) or for 20 min (in the case of the animals used to study the axon initial segment [59]) in the same fixative at 4 ºC. They were then washed three times in 0.1 N PB.

### Brain tissue sectioning

For immunohistochemical determinations, brains were included in a 10% sucrose-4% agarose solution [63–65] after being rinsed, and 50 µm-thick coronal sections were obtained on a Leica VT1200S vibratome. Series of brain slices were randomly made up of one section from every ninth. For each series of sections, the sampling probability was 1/8. The brain sections were immediately stored at – 20 ºC in 24-well plastic plates filled with a cryopreservative solution (30% polyethylene glycol; 10% 0.2 N PB; 30% glycerol; 30% bi-distilled water). For the electron microscopy studies, 100-µm sections were obtained on a vibratome.

### Immunohistochemistry

Briefly, slices were rinsed in 0.1 N PB at RT. Triple IHC was performed as described previously [35]. The incubation buffer for all primary (Supplementary Table T1) and secondary (Supplementary Table T2) antibodies contained 1% Triton X-100 and 1% BSA diluted in 0.1 N PB. Incubation with all the primary antibodies was performed under gentle shaking at 4 ºC between 48 and 72 h. To detect the binding of primary antibodies, the sections were subsequently incubated with Alexa®-coupled fluorescent secondary antibodies for 24 h at 4 ºC. After this incubation, the sections were rinsed three times in 0.1 N PB and counterstained for 10 min with DAPI (1:5000) to label nuclei. They were then mounted on gelatin-coated glass slides. A non-commercial anti-fading mounting medium (33% glycerol and 7.5% mowiol, prepared in Tris–HCl 0.2 M pH = 8.5) was used for embedding the sections.

### Electron microscopy

#### Sample preparation

After sectioning, slices were prepared as previously described [53]. Briefly, GFP-expressing cells were injected with 5% aqueous Lucifer Yellow (Sigma). The slices were then incubated with 2.8 mM 3,3′-Diaminobenzidine (DAB) and 6 mM potassium cyanide, and then irradiated under conventional epifluorescence using a 75-W Hg lamp. The latter step causes the photoconversion of DAB into an electron-dense residue. Slices were then exposed to O/N postfixation in a solution of 3% glutaraldehyde and processed for electron microscopy. Next, 45–200 serial sections of 40 nm thickness were obtained under an Ultracut E ultramicrotome. Labeled dendritic segments were sectioned longitudinally.

#### Imaging, segmentation, and reconstruction of inhibitory synapses

Dendritic segments were imaged under a Megaview III camera mounted on a JEOL-100CXII electron microscope at a 19,000 × magnification. Images were contrasted and stitched using Adobe Photoshop and aligned using the Align software (provided by J. Fiala, Boston University). The components of each inhibitory synapse (namely the axon terminal, adult-born dentate granule cell dendrite, presynaptic and postsynaptic densities, and synaptic vesicles) were individually identified following the morphological criterial described in [66, 67] and then segmented using the *Drawing* tools of *3dmod* plugin for *IMOD* software. The contour of each component was drawn in all the images. The small-fold procedure was used to determine section thickness and an average value of 34 ± 1.33 nm was used for reconstruction. The 3D reconstruction of an inhibitory synapse made onto a 4-week-old newborn dentate granule cell shown in Fig. 1F was performed using *Model View* tool for IMOD software.

#### Quantification of presynaptic vesicles

Serial electron microscopy images were used to manually count the number of presynaptic vesicles. Each synaptic vesicle was classified into distinct pools (readily-releasable, recycling, or reserve) on the basis of its position within the axon terminal and its putative function at the inhibitory synapse, as previously described [68]. The total volume (reference volume) of the axonal terminal was defined as the addition of the area displayed by this structure in each image multiplied by the thickness of the section. The total number of presynaptic vesicles was divided by the reference volume, and the density (number of synaptic vesicles/µm^3^) of presynaptic vesicles belonging to each category was calculated.

### Morphology of newborn dentate granule cells

At least 30 randomly selected adult-born dentate granule cells, of each age and genotype, transduced with a Gephyrin:GFP-encoding retrovirus were reconstructed in a LSM800 Zeiss confocal microscope (40X oil immersion objective). Confocal stacks of images were obtained (XY dimensions: 199.66 μm; 0.8 × zoom; Z-axis interval: 0.5 µm), and Z-projections were analyzed to determine the total dendritic length and dendritic arbor branching (Sholl´s analysis). All cells were traced using *NeuronJ* plugin for *Fiji* software (ImageJ,v. 1.50e, NIH, Bethesda, MD, USA, http://rsb.info.nih.gov/ij). Sholl´s analysis was performed using the plugin *ShollAnalysis* for *Fiji* [34, 69].

### Cell counts

To determine the density of dentate gyrus Parvalbumin^+^ interneurons, five stacks of images of the dentate gyrus per animal were obtained using an LSM710 Zeiss confocal microscope (25X oil immersion objective; XY dimensions: 425.10 µm; Z-interval: 1.4 µm). The density of Parvalbumin ^+^ cells was estimated by unbiased stereology methods based on the use of the physical dissector method adapted to confocal microscopy, as previously described [63–65, 70]. Briefly, an area containing the region of interest (namely the Hilus, and/or the granule cell layer plus the subgranular zone) was traced on the DAPI channel of each confocal stack of images using the freehand drawing tool of *Fiji*, and the area of each of these structures was then calculated. Areas were multiplied by the z-thickness of the stack to calculate the reference volume [63, 64]. Next, Parvalbumin^+^ cells were counted on individual planes. The total number of positive cells was then divided by the reference volume of the stack, and the density of positive cells (number of cells/mm^3^) was calculated. Supplementary Fig. S3I–K shows the density of Parvalbumin^+^ cells regardless of their positioning. Supplementary Fig. S5 shows regional Parvalbumin^+^ interneuron densities.

### Analysis of Gephyrin^+^ postsynaptic clusters

Confocal stacks of images containing adult-born dentate granule cells transduced with a Gephyrin:GFP-encoding retrovirus were obtained under an LSM800 Zeiss confocal microscope (63X oil immersion objective; 3 × zoom; image XY dimensions: 33.80 μm; Z-axis interval: 0.2 µm). At least 30 dendritic segments of each order (1st, 2nd, 3rd, 4th, and 5th), and 30 somas per genotype and cell age were analyzed. The dendritic length of each segment or the perimeter of the soma was measured on the GFP channel, and the number and area of Gephyrin^+^ postsynaptic clusters were analyzed using the semi-automatic *Particle Analyzer* plugin for *Fiji*. Next, the density of Gephyrin^+^ postsynaptic clusters was estimated by dividing the number of Gephyrin^+^ clusters by the segment length, as previously described [34]. To compare the density and area of Gephyrin^+^ clusters in WT and GSK-3β-OE mice, data obtained from dendrites of the 2nd, 3rd, 4th, and 5th orders were pooled. Therefore, Fig. 2A, B and Supplementary Fig. S3C, D show the density and area of Gephyrin^+^ clusters in dendrites of 1st (Fig. 2A and Supplementary Fig. S3C) and higher (Fig. 2B and Supplementary Fig. S3D) branching orders separately.

### Colocalization analyses between Bassoon and Gephyrin

A minimum of 50 images per genotype (63X oil immersion objective; 3 × zoom; image XY dimensions: 33.80 μm; Z-axis interval: 0.3 µm) of adult-born dentate granule cells retrovirally labeled with Gephyrin:GFP were obtained under an LSM900 Zeiss confocal microscope coupled to an *Airyscan* super-resolution microscopy module. Colocalization between the presynaptic marker Bassoon and the postsynaptic scaffolding protein Gephyrin was examined using the *Just Another Co-localization Plugin (JACoP)* for *Fiji*. M1 Mander´s coefficient was calculated and represented in the graphs.

### Percentage of newborn dentate granule cells surrounded by Parvalbumin^+^ interneurons

A minimum of 50 randomly selected adult-born dentate granule cells of each genotype transduced with a Gephyrin:GFP-encoding retrovirus were imaged using an LSM900 Zeiss confocal microscope (63X oil immersion objective; 3.5 × zoom; image XY dimensions: 27.62 μm; Z-axis interval: 0.15 µm) coupled to an *Airyscan* super-resolution microscopy module. The presence of Parvalbumin^+^ interneurons was detected on each plane. The percentage of adult-born dentate granule cells surrounded by Parvalbumin^+^ interneurons was calculated by dividing the total number of dentate granule cells surrounded by Parvalbumin^+^ interneurons by the total number of adult-born dentate granule cells analyzed.

### Parvalbumin^+^ volume surrounding each Gephyrin^+^ cluster

We obtained 35–60 stacks containing randomly selected adult-born dentate granule cells, of each genotype and cell age, transduced with a Gephyrin:GFP-encoding retrovirus under an LSM900 Zeiss confocal microscope (63X oil immersion objective; 3.5 × zoom; image XY dimensions: 27.62 μm; Z-axis interval: 0.15 µm) coupled to an *Airyscan* super-resolution microscopy module. At least 30 Gephyrin^+^ clusters located at each subcellular compartment (1st order dendrite, soma (total, apical, and basal domains), and axon) were analyzed per genotype and cell age. An invariant threshold for Gephyrin fluorescence intensity was first established on the green channel to identify individual Gephyrin^+^ clusters. Next, the *Dilate* utility for *Fiji* was applied to expand the cluster contour in the X and Y dimensions by 5 pixels. Subsequently, the area of the cluster was subtracted to establish a ring of fixed diameter surrounding each cluster on each plane. The *3D ROI Manager* plugin was used to calculate the reference volume by summing the area of the rings of each plane. An invariant threshold for Parvalbumin fluorescence intensity (grey channel) was then established, and the Parvalbumin^+^ area over the threshold was calculated in each plane by means of *Fiji*. The Parvalbumin^+^ volume over the threshold was calculated by summing the positive area of each plane and dividing by the reference volume to estimate the Parvalbumin^+^ volume surrounding each Gephyrin^+^ cluster (Supplementary Fig. S1C).

### Proportion of the soma and axon occupied by Gephyrin^+^ clusters

The soma and axon of at least 30 adult-born dentate granule cells, of each genotype and cell age, labeled with a Gephyrin:GFP-encoding retrovirus were obtained under an LSM800 Zeiss confocal microscope (63X oil immersion objective; 3 × zoom; image XY dimensions: 33.80 μm; Z-axis interval: 0.2 µm). The total Gephyrin^+^ area was defined as the sum of the area of all the individual Gephyrin^+^ clusters found either in the soma or in the axon. The percentage of the soma occupied by Gephyrin^+^ clusters was then calculated by dividing the total Gephyrin^+^ area by the volume of the soma. Separate analyses were performed for total, basal, and apical domains of the soma. Moreover, the proportion of each axon occupied by Gephyrin^+^ clusters was calculated by dividing the total Gephyrin^+^ area by the length of the axonal segment.

### Fluorescence intensity of Lectin^+^ and Aggrecan^+^ Perineuronal nets

To study the fluorescence intensity of perineuronal nets surrounding dentate gyrus Parvalbumin^+^ interneurons, 40–50 stacks of images per genotype were obtained under an LSM710 Zeiss confocal microscope (25X oil immersion objective; XY dimensions: 425.10 µm; Z-interval: 1.4 µm). An invariant threshold was set in *Fiji* to allow delineation of the soma of each Parvalbumin^+^ interneuron on the green channel. Next, the *Dilate* utility was applied to expand the soma contour in the X and Y dimensions by 3 pixels. Subsequently, the area of the soma was subtracted to establish a ring of fixed diameter surrounding the cell on each plane. The *3D ROI Manager* plugin was used to calculate the reference volume and mean intensity of perineuronal marker staining (Lectin in the red channel, and Aggrecan in the grey channel). Independent calculations were made for each dentate gyrus subregion of interest, namely the granule cell layer, subgranular zone, and Hilus.

### Percentage of Parvalbumin^+^ interneurons surrounded by perineuronal nets

We obtained 40–50 stacks of dentate gyrus images per genotype under an LSM710 Zeiss confocal microscope (25X oil immersion objective; XY dimensions: 425.10 µm; Z-interval: 1.4 µm). A ring surrounding the soma of Parvalbumin^+^ interneurons was set to determine Lectin and Aggrecan fluorescence intensity, as previously described. Next, these interneurons were classified on the basis of the fluorescence intensity detected for each marker. The percentage of Parvalbumin^+^ interneurons falling into the distinct intensity categories is shown in the graphs.

### Electrophysiology

Acute slices for electrophysiological recordings were prepared from 7- and 14-week-old female mice of each genotype, 1 and 8 weeks after Gephyrin:GFP-encoding retrovirus injection, respectively. The brain was rapidly removed from the skull, and coronal slices (300 μm) containing the dorsal hippocampus were cut on a Leica VT1200S vibratome at 4 °C immersed in a solution containing 234 mM sucrose, 11 mM glucose, 26 mM NaHCO_3_, 2.5 mM KCl, 1.25 mM NaH_2_PO_4_,10 mM MgSO_4_, and mM 0.5 CaCl_2_ (equilibrated with 95–5% CO_2_). The slices were incubated for more than 1 h at room temperature (22–24 °C) in an oxygenated artificial cerebrospinal fluid (ACSF) containing 126 mM NaCl, 26 mM NaHCO_3_, 2.5 mM KCl, 1.25 mM NaH_2_PO_4_, 2 mM MgSO_4_, 2 mM CaCl_2_ and 10 mM glucose (pH 7.4). They were then transferred to a recording immersion chamber and perfused with gassed ACSF.

Electrophysiological recordings of dentate granule cells were performed using the whole-cell configuration of the patch-clamp technique. Recordings were obtained at 30–32 °C from dentate granule cells visually identified by means of an infrared video and a fluorescence microscopy equipped with a 488-nm wavelength filter. Only adult-born dentate granule cells transduced by Gephyrin:GFP-encoding retroviruses showed visible Gephyrin^+^ clusters in the soma. Developmentally generated dentate granule cells were not transduced with the Gephyrin:GFP retroviruses and were thus GFP-negative. The position of newborn dentate granule cells within the granule cell layer changes with age [56]. One-week-old adult-born dentate granule cells were located at the SGZ, whereas 8-week-old newborn dentate granule cells occupied upper positions of the granule cell layer and showed larger Gephyrin^+^ clusters in the soma.

Patch electrodes had resistances of 3–5 MΩ when filled with the internal solution composed of 70 mM K-Gluconate, 68 mM KCl, 10 mM HEPES, 4 mM EGTA, 4 mM MgATP and 4 mM QX-314 bromide, pH 7.3 adjusted with KOH (290 mOsm). GABA receptor-mediated spontaneous miniature inhibitory postsynaptic currents were registered by clamping neurons at – 70 mV, adding Kynurenic acid (2 mM) to block glutamatergic transmission and TTX (1 μM) to the ACSF. Signals were amplified using a Multiclamp 700B patch-clamp amplifier and digitized using a Digidata 1550B (Axon Instruments, USA), sampled at 20 kHz, filtered at 10 kHz, and stored on a PC using Clampex 10.7 (Axon Instruments). Series resistance was monitored regularly during recordings to assess stable recording conditions. Miniature inhibitory postsynaptic currents were analyzed using pClamp (Axon Instruments) and custom-written software (Detector, courtesy J. R. Huguenard, Stanford University). Briefly, individual events were detected with a threshold-triggered process from a differentiated copy of the recordings. For each cell, the detection criteria (threshold and duration of trigger for detection) were adjusted to rule out slow membrane fluctuations and electric noise while allowing maximal discrimination of miniature inhibitory postsynaptic currents. Detection frames were regularly inspected to ensure that the detector was working properly.

### Morphometric analysis of the axonal initial segment of newborn dentate granule cells

To analyze the morphometric parameters of the axon initial segment of dentate granule cells, we measured the expression of the axon initial segment marker Ankyrin G in the axons of retrovirally labeled cells [59]. Given that axonal projections show a regular thickness and are markedly thinner than dendrites, they were identified on the basis of morphological features [56]. A minimum of 30 cells per genotype and cell age were reconstructed in a Nikon A1R + confocal microscope (40X oil immersion objective; 0.8 × zoom; image XY dimensions: 318.51 µm; Z-interval: 2 µm). The expression of Ankyrin G throughout axonal projections was confirmed in each plane of the Z-stacks using the *Orthogonal Views* tool in *Fiji* software. Ankyrin G expression was used to determine the starting and ending points of the axon initial segment. First, to determine the axon initial segment starting point, the axon was followed in the green channel, and the first point showing colocalization between GFP and Ankyrin G was identified. The distance between the axonal hillock and the starting point of Ankyrin G expression was then measured on Z-projection images using the freehand drawing tool of *Fiji* and was subsequently referred to as the axon initial segment starting point. The distance between the axon initial segment starting point and the end point of Ankyrin G expression was measured using a similar methodology, and this parameter was denoted axon initial segment length.

### Synaptophysin (Syn)^+^ area of mossy fiber terminals of newborn dentate granule cells

A minimum of 10–15 stacks of images per experimental condition and region were obtained in an inverted LSM710 Zeiss confocal microscope (63X oil immersion objective; XY dimensions: 103.81 µm; Z-interval: 0.4 µm). To measure the Syn^+^ area of mossy fiber terminals, colocalization between GFP and Syn was analyzed by means of the Just Another Co-localization (JACoP) plugin for *Fiji*. M2 Mander´s coefficient was calculated and represented in the graphs.

### Novel location preference test

The novel location preference paradigm is highly sensitive to variations in adult hippocampal neurogenesis [71]. The test was performed on a square (45 × 45 cm), constantly illuminated, open-field methacrylate arena on three consecutive days. Each day, animals were subjected to a single 10-min trial. On the first day, they were habituated and allowed to explore the arena. On the second day (sample phase), two identical objects were placed symmetrically in the central part of the arena. On the third day (test phase), one of the objects (novel-located object) was moved to a peripheral position, while the other remained unaltered. Animal performance was recorded and automatically analyzed by Any-maze software (Stoelting, USA). The time exploring each object was measured. Memory index (time exploring novel-located object/time exploring novel + unaltered object) and total exploratory time (time exploring novel + unaltered object) are shown in the graphs.

### Statistical analyses

Statistical analyses were conducted using GraphPad Prism 9 software (GraphPad.v.9.5.0 (730), 2022; GraphPad Software, LLC). The normality of sample distribution was assessed by means of a Kolmogorov–Smirnov test (data distribution was considered to be normal for *p* > 0.05). Atypical data were identified by the same software and extreme outlier values were eliminated when necessary. To compare two experimental groups, either a Student *t*-test or a one-way ANOVA followed by a post hoc Tukey test was used in those cases showing normal sample distribution. For those cases in which normality could not be assumed, a nonparametric test (either a Mann–Whitney *U* test or a Kruskal–Wallis test followed by a post hoc Dunn test) was performed. For comparisons between more than two experimental groups, a two-way ANOVA test was used. Fischer LSD post hoc analysis (Tukey or Bonferroni) was used to compare the differences between individual groups. Data from Sholl’s analysis were analyzed by a repeated measures ANOVA test [35]. The percentage of adult-born dentate granule cells surrounded by Parvalbumin^+^ interneurons was studied by means of a Chi-square test. Graphs represent mean values ± SEM. A 95% confidence interval was used for statistical comparisons. The detailed results of statistical comparisons are included in Supplementary file7. In all the main and Supplementary Figures, blue asterisks indicate statistically significant differences between genotypes. In Supplementary Figs. S3J–K and S5, black asterisks indicate differences between staining intensity categories. In the remaining main and Supplementary Figures, black asterisks indicate differences between cell ages.

## Results

### Maturation of inhibitory synapses made onto newborn dentate granule cells of wild-type (WT) mice

To longitudinally study the inhibitory innervation of newborn dentate granule cells, we stereotaxically injected a Gephyrin:GFP-encoding retrovirus (Fig. 1A, B and Supplementary Fig. S1A, B and D) into the dentate gyrus of adult C57 BL6JOla/Hsd mice, following a similar strategy to that previously used to visualize the excitatory postsynaptic densities [34, 35, 60, 72] and presynaptic active zones [35, 60, 73] of these cells. The animals were sacrificed at distinct post-injection times following the experimental design depicted in Supplementary Fig. S1B. The endogenous fluorescence of the retrovirus (green) enabled visualization of Gephyrin bound to the postsynaptic densities of inhibitory synapses, whereas the use of an anti-GFP primary antibody combined with a red secondary antibody enhanced visualization of the intracellular trafficking of the free protein, thereby allowing the identification of cell morphology (Supplementary Fig. S1D). It should be noted that no co-localization between PSD95 and Gephyrin was detected in cells co-transduced in vivo by PSD95- and Gephyrin-encoding retroviruses (Supplementary Fig. S1E). This observation suggests that the viral-mediated expression of these fusion proteins did not interfere with their subcellular localization. *Airyscan* super-resolution microscopy showed marked apposition between Bassoon^+^ presynaptic terminals and newborn dentate granule cell Gephyrin^+^ postsynaptic clusters (Supplementary Figure S1F). The presence of inhibitory synaptic contacts onto newborn dentate granule cells was further confirmed by transmission electron microscopy (Fig. 1C – F and Supplementary Fig. S2). The presence of symmetric presynaptic and postsynaptic densities, clearly defined synaptic clefts, and at least 3 synaptic vesicles within 150 nm of the presynaptic membrane were used to identify inhibitory synapses, as previously described [66, 67].

To quantitatively assess the abundance and characteristics of inhibitory synaptic contacts made onto newborn dentate granule cells throughout the maturation of these cells, we first determined the density and area of Gephyrin^+^ clusters at distinct subcellular localizations. The density of these clusters increased with cell age in dendrites of 1st (*K*_4, 140_ = 76.08; *p* < 0.001), 2nd (*K*_4, 141_ = 94.37; *p* < 0.001), 3rd (*K*_4, 150_ = 108.80; *p* < 0.001), 4th (*K*_3, 123_ = 63.73; *p* < 0.001), and 5th (K_3, 122_ = 68.94; *p* < 0.001) orders, as well as in the soma (*K*_4, 155_ = 41.20; *p* < 0.001) and axon (*K*_4, 146_ = 75.53; *p* < 0.001), reaching a *plateau* in several subcellular localizations at ~ 3–4 weeks (Fig. 1G–I and Supplementary file7). Similarly, the area of Gephyrin^+^ clusters varied in the dendrites of 1st (*K*_4, 85_ = 27.25; *p* < 0.001), 2nd (*F*_4, 95_ = 35.95; *p* < 0.001), 3rd (F_4, 104_ = 38.46; p < 0.001), 4th (*K*_3, 113_ = 55.79; *p* < 0.001), and 5th (*K*_3, 106_ = 45.76; *p* < 0.001) orders, and in the soma (*K*_4, 131_ = 38.08; *p* < 0.001), and axon (*K*_4, 123_ = 28.55; *p* < 0.001) during dentate granule cell maturation (Fig. 1J**–**L). Accordingly, the proportion of the somatic (*K*_4, 155_ = 80.29; *p* < 0.001) and axonal (*K*_4, 125_ = 73.23; *p* < 0.001) compartments occupied by Gephyrin^+^ clusters also increased with cell age (Supplementary Fig. S1G–J). Given that Parvalbumin^+^ interneurons thoroughly innervate the perisomatic compartment of newborn dentate granule cells and are key players in regulating their maturation [15], we next examined whether Gephyrin^+^ clusters of the latter cells were specifically contacted by Parvalbumin^+^ terminals (Fig. 1M). We observed progressive apposition between Parvalbumin^+^ terminals and Gephyrin^+^ clusters located at the 1st order dendrites (*K*_4, 141_ = 13.78; *p* = 0.008) (Supplementary Fig. S1K), soma (*K*_4, 141_ = 52.26; *p* < 0.001) (Supplementary Fig. S1L–N), and axon (K_4, 223_ = 41.49; *p* < 0.001) (Supplementary Fig. S1O) of newborn dentate granule cells during their maturation. In agreement with previous data [15, 16], no evident apposition between Parvalbumin^+^ terminals and Gephyrin^+^ clusters was observed in distal dendrites.

Taken together, these data support the progressive maturation of inhibitory synapses during adult hippocampal neurogenesis, as shown by electrophysiological recordings [15]. Furthermore, these results point to the presence of inhibitory postsynaptic densities of very immature 1-week-old dentate granule cells and their maturation time course.

### Impaired maturation of inhibitory synapses made onto newborn dentate granule cells of a mouse model of Alzheimer´s disease (AD)

Impaired excitatory innervation of adult-born dentate granule cells has been consistently reported in mouse models of AD [34, 74]. However, the extent to which the inhibitory innervation of these cells is also altered remains unexplored to date.

We first confirmed previous data showing aberrant morphological development [34] (namely altered total dendritic length (Supplementary Fig. S4B, D, F, H, J) and number of crossings in Sholl´s analysis (Supplementary Fig. S4A, C, E, G, and I)) in a mouse model of AD that overexpresses GSK-3β [34], one of the key players in this condition. We found that GSK-3β overexpression altered the density of Gephyrin^+^ clusters in dendrites of 1st (Genotype: *F*_1, 292_ = 4.01; *p* = 0.046; Cell age: *F*_4, 292_ = 59.23; *p* < 0.001) and higher (Genotype: *F*_1, 1067_ = 4.93; *p* = 0.027; Cell age: *F*_4, 1067_ = 297.20; *p* < 0.001) branching order dendrites across newborn dentate granule cell maturation (Fig. 2A, B). Moreover, the area of Gephyrin^+^ clusters was altered in the dendrites of 1st (Genotype: F_1, 187_ = 4.73; *p* = 0.031; Cell age: *F*_4, 187_ = 8.96; *p* < 0.001) and higher (Genotype: *F*_1, 855_ = 8.41; *p* = 0.040; Cell age: *F*_4, 855_ = 74.10; *p* < 0.001) branching order dendrites, and axon (Genotype: *F*_1, 216_ = 22.24; *p* < 0.001; Cell age: *F*_4, 216_ = 17.35; *p* < 0.001) of GSK-3β-OE newborn dentate granule cells throughout their maturation (Supplementary Fig. S3C, D and F).

In light of the observed increased apposition between Bassoon^+^ presynaptic terminals and GSK-3β-OE newborn dentate granule cell Gephyrin^+^ postsynaptic clusters (*U* = 1144; *p* = 0.004) (Fig. 2D), we next addressed whether increased innervation by Parvalbumin^+^ interneurons occurred. Despite unchanged numbers of Parvalbumin^+^ interneurons (*t* = 0.551; *p* = 0.589) (Supplementary Figs. S3I and S5A–C), GSK-3β-OE mice showed an increased number of immature dentate granule cells whose soma was surrounded by Parvalbumin^+^ interneurons (Genotype: *χ*^2^_1, 13.13_ = 3.623; *p* < 0.001) (Fig. 2E). Accordingly, increased Parvalbumin^+^ volume surrounded Gephyrin^+^ clusters located at the soma of newborn dentate granule cells (Genotype: *F*_1, 655_ = 18.82; *p* < 0.001; Cell age: *F*_4, 655_ = 9.04; *p* < 0.001) (Fig. 2F, G), although a similar trend was not observed in the 1st order dendrites (Genotype: *F*_1, 312_ = 0.80; *p* = 0.371; Cell age: *F*_4, 312_ = 9.09; *p* < 0.001) or the axon (Genotype: *F*_1, 388_ = 2.367; *p* = 0.125; Cell age: *F*_4, 388_ = 9.55; *p* < 0.001) of these cells (Supplementary Fig. S3G, H, and Supplementary file7).

Of note, the presence of perineuronal nets was altered in Parvalbumin^+^ interneurons of GSK-3β-OE mice, as reflected by the reduced intensity of Lectin^+^ (*U* = 11; *p* = 0.009) (Fig. 2H) and Aggrecan^+^ (t = 5.021; p < 0.001) (Fig. 2I) perineuronal nets surrounding Parvalbumin^+^ interneurons located in distinct regions of the dentate gyrus (Fig. 2J–K, Supplementary Fig. S5D–O and Supplementary file7). Accordingly, higher numbers of Parvalbumin^+^ cells were devoid of Lectin^+^ (*p* < 0.001) (Supplementary Figure S3J) and Aggrecan^+^ (*p* < 0.001) (Supplementary Fig. S3K) perineuronal nets. The absence of perineuronal nets might alter Parvalbumin^+^ interneuron excitability [75], as well as expose these cells to toxic environmental cues [21], thereby leading to aberrant synaptic transmission and resulting in the altered inhibition of newborn dentate granule cells.

### Decreased functional output from newborn dentate granule cells in a mouse model of Alzheimer´s disease (AD)

We first tested whether the inhibitory innervation of newborn dentate granule cells was functionally altered in GSK-3β-OE mice by performing whole-cell patch-clamp recordings of 8-week-old retrovirally labeled newborn dentate granule cells (Fig. 3A). Despite not detecting changes in the frequency of miniature inhibitory postsynaptic currents (Fig. 3E), we found increased miniature inhibitory postsynaptic current Amplitude (*p* = 0.0406) (Fig. 3F) and slope (*p* = 0.0221) (Fig. 3G) in these mice. This observation is compatible with an enhanced inhibitory innervation of newborn dentate granule cells. Similar effects were not observed in developmentally generated dentate granule cells (miniature inhibitory postsynaptic current Frequency: *p* > 0.9999; Amplitude: *p* = 0.4023; Slope: *p* = 0.7579) (Fig. 3E**–**G) or in 1-week-old newborn dentate granule cells (miniature inhibitory postsynaptic current Frequency: (*p* = 0.2494); Amplitude: (*p* > 0.9999); Slope: (*p* > 0.9999) (Supplementary Fig. S3L–Q). It should be noted that GSK-3β is not yet overexpressed in the latter cells and that they may escape from phasic GABAergic innervation [7, 12]. In light of this putatively enhanced inhibitory innervation in GSK-3β-OE mice, we wondered whether these alterations reduce newborn dentate granule cell synaptic output. To address this notion, we first stereotaxically injected a Venus-encoding retrovirus to study the axon initial segment of newborn dentate granule cells [59]. GSK-3β-OE mice showed shorter axon initial segment (Genotype: *F*_1, 166_ = 47.11; *p* < 0.001; Cell age: *F*_2, 166_ = 17.94; *p* < 0.001) (Fig. 3H) whose starting points were located farther from the soma (Genotype: *F*_1,167_ = 11.62; *p* < 0.001; Cell age: *F*_2,167_ = 1.94; *p* = 0.147) (Fig. 3I). This finding, which might be related to the dendritic alterations observed [76], points to decreased synaptic output from newborn dentate granule cells. Accordingly, the stereotaxic injection of a Synaptophysin:GFP-encoding retrovirus showed that, in GSK-3β-OE mice, the active area of newborn dentate granule cell mossy fiber terminals occupied by Syn^+^ presynaptic clusters (Fig. 3L–M) tended to be reduced in the CA3 (Genotype: *F*_1, 98_ = 3.688; *p* = 0.058; Cell age: *F*_2, 98_ = 27.96; *p* < 0.001) (Fig. 3N) and was decreased in the CA2 (Genotype: *F*_1, 65_ = 12.52; *p* < 0.001; Cell age: *F*_2, 65_ = 24.10; *p* < 0.001) (Fig. 3O) hippocampal subfields as compared to the WT. We next tested whether the putatively altered synaptic output is paralleled by functional impairments in pattern separation capacity. In this regard, despite showing increased total exploration time during the 3rd day (*t* = 3.774; *p* = 0.002) (Fig. 3P), GSK-3β-OE mice exhibited a reduced Memory index in the novel location preference test (*t* = 2.352; *p* = 0.033) (Fig. 3Q). It should be noted that this pattern-separation task has been related to newborn dentate granule cell function [4]. Taken together, these data indicate that the aberrant inhibitory innervation of GSK-3β-OE newborn dentate granule cells is accompanied by marked functional impairments in these cells.

## Discussion

The addition of new neurons to the hippocampus provides enhanced plasticity to this structure and contributes to the cognitive reserve during aging. Adult hippocampal neurogenesis is compromised in patients with neurodegenerative and psychiatric diseases and in animal models of these conditions [64, 65, 77, 78]. However, the molecular mechanisms underlying hippocampal impairments in patients with these conditions remain to be fully elucidated. In this regard, several abnormalities related to inhibitory synapses have been detected in patients with neurological disorders [35]. GABA supports the activity-dependent growth of dendrites and synapses during adult and developmental neurogenesis. Moreover, tonic and phasic GABAergic neurotransmission orchestrate the global hippocampal function. In the dentate gyrus, distinct populations of interneurons span all layers from the outer molecular layer to the Hilus, thereby receiving excitatory inputs from all the major dentate gyrus afferents and, thus, being able to provide both feedforward and feedback compartment-selective inhibition to dentate granule cells and other interneurons [18]. Moreover, beyond direct synaptic inhibition, interneurons can also provide GABA *spillover* to the extracellular space [13, 18]. In the precise context of adult hippocampal neurogenesis, the stimulation of local interneurons induces the depolarization of neural stem cells (NSCs) and regulates their quiescence/activation ratio [79, 80]. Moreover, tonic GABA *spillover* regulates the early maturation of newborn dentate granule cells, and GABA-induced depolarization coordinates the establishment of functional glutamate- and GABA-mediated synapses onto these cells [7]. Due to high intracellular chloride concentration, GABAergic transmission is depolarizing in immature dentate granule cells but becomes inhibitory at 3–4 weeks of age [10]. Compared to mature dentate granule cells [14, 81], their immature counterparts show GABAergic dynamics characterized by slower kinetics and lower frequency, which contribute to their greater synaptic activation [82]. In this regard, although Parvalbumin^+^ interneurons innervate dentate granule cells early in their development [16], synaptic contacts made onto newborn dentate granule cells require several weeks to achieve full maturation [15]. In fact, the number and size of inhibitory postsynaptic clusters located at distinct subcellular localizations markedly rise during cell maturation. Moreover, the surrounding of these clusters by Parvalbumin^+^ presynaptic terminals progressively increases with cell age. Taken together, these data point to sustained, albeit slow, structural remodeling of inhibitory synapses during adult hippocampal neurogenesis. Gephyrin cannot form inhibitory postsynaptic densities in the absence of neurotransmitter receptor assemblies [32, 33]. Therefore, the presence of Gephyrin^+^ postsynaptic clusters and miniature inhibitory postsynaptic currents in 1-week-old newborn dentate granule cells point to functional GABAergic transmission onto these cells. These stimuli might contribute to the well-known early trophic effects exerted by GABA on very immature dentate granule cells. Moreover, these data support the notion that the formation of inhibitory postsynaptic densities responsive to GABAergic stimuli precedes, by several weeks, the full integration of newborn dentate granule cells into hippocampal inhibitory circuits. Our morphometric data suggest a stabilization of Gephyrin^+^ postsynaptic cluster density at ~ 3–4 weeks of cell age in various subcellular compartments, whereas other cell compartments (such as the axon initial segment) might require longer to achieve stability. In fact, at the ultrastructural level, we did not observe changes in the number of presynaptic vesicles belonging to the docked, proximal, or distal pools after 4 weeks of maturation in dendritic inhibitory synapses (Supplementary Fig. S2).

Several molecular pathways regulate the formation of inhibitory synapses in the adult brain [83–85] (reviewed in [86]). Of interest, some of these pathways converge on the regulation of GSK-3β activity. This kinase plays a pivotal role in various neurodegenerative diseases, including AD and Tauopathies, and it is also a master regulator of adult hippocampal neurogenesis [87]. In fact, GSK-3β-OE mice present early adult hippocampal neurogenesis impairments [34, 46, 48–50]. These animals start to show a depletion of neural stem cell niches and reduced survival of newly generated neurons at ~ 2–3 months of age [48, 50]. At the same age, they also present reduced dentate gyrus volume and increased apoptosis and astrocytosis at two months of age [49]. Moreover, GSK-3β overexpression triggers maturation abnormalities in newly generated neurons, including the lengthening of their immaturity period [48] and the acquisition of an aberrant morphological phenotype characterized by the presence of several primary apical dendrites (named the “*V-shape*” phenotype) [34]—a phenomenon also observed in patients with AD [88] and frontotemporal dementia [35]. Moreover, the overexpression of this kinase leads to functional impairments in newborn dentate granule cells, as GSK-3β-OE neurons show a reduced number and size of postsynaptic clusters at excitatory synapses [34]. Our data point to an in vivo role of GSK-3β in regulating the establishment and maturation of inhibitory synapses during adult hippocampal neurogenesis. Similar to other actions of GSK-3β on adult hippocampal neurogenesis [48], a regulatory role on inhibitory synapses is likely to be exerted throughout the entire life of newborn dentate granule cells. This hypothesis is supported by the fact that, in GSK-3β-OE mice, these cells systematically display altered numbers of inhibitory synapses and aberrant area of Gephyrin^+^ clusters in distinct subcellular compartments during their maturation. These structural abnormalities are expected to impact the overall plasticity and stability of inhibitory synapses made onto newborn dentate granule cells. In fact, these cells show faster miniature inhibitory postsynaptic currents (Fig. 3B–D and F, G), which correlate with the shortening and distancing of the axon initial segment from the soma (Fig. 3H–K) and reduced synaptically active area in the CA3 and CA2 regions (Fig. 3L–O). Taken together, these results point to altered (putatively enhanced) inhibitory innervation and reduced synaptic activity of GSK-3β-OE newborn dentate granule cells. Moreover, these findings are in agreement with previous in vitro reports indicating that GSK-3β regulates inhibitory synapse composition and abundance through Gephyrin Ser270 phosphorylation [52]. This phosphorylation alters the density and size of Gephyrin^+^ and GABA_A_ receptor^+^ clusters [52, 89] and modifies the amount of Gephyrin *per* cluster [52, 89]. Conversely, GSK-3β inhibitors limit the size of Gephyrin^+^ postsynaptic clusters and Gephyrin availability [52]. Of note, the putatively increased inhibitory innervation of newborn dentate granule cells observed in GSK-3β-OE mice might lead to decreased synaptic output at the mossy fiber tract [90] and may also contribute to the observed functional deficits in pattern separation capacity (Fig. 3Q) and global hippocampal dysfunction [44, 45, 47, 90].

The well-known deleterious in vivo effects of GSK-3β overexpression on adult hippocampal neurogenesis [34, 48] might also per se impact dentate gyrus interneuron functioning through the modulation of perineuronal nets. Of note, the formation of the latter structures is regulated by experience-dependent plasticity, and reduced excitatory inputs result in decreased perineuronal net formation [20]. In fact, the presence of perineuronal nets makes Parvalbumin^+^ interneurons more likely to be contacted by adult-born dentate granule cells, and a reduction in the number of adult-born dentate granule cells leads to a dramatic drop in the number of Parvalbumin^+^ interneurons with perineuronal nets and an overall decrease in perineuronal net fluorescence intensity [20]. Therefore, a reduction in adult hippocampal neurogenesis caused by GSK-3β overexpression [34, 48] might have dramatic consequences on the plasticity of dentate gyrus inhibitory circuits through the modulation of the function and stability of perineuronal nets. On the other hand, Parvalbumin^+^ interneuron alterations might be related to *non-cell-autonomous* mechanisms triggered by the overexpression of this kinase. In this regard, the remarkable hippocampal neuroinflammation previously described in GSK-3β-OE mice [46] might further contribute to the loss of perineuronal nets in Parvalbumin^+^ interneurons in the dentate gyrus. Crapser et al. observed that the loss of perineuronal nets in the subiculum of AD patients and animal models of this disease directly involves microglia activation [26]. Microglia release proinflammatory cytokines (e.g., TNF-α and IL-1β), reactive oxygen species, and matrix metalloproteinases (MMPs), the latter having the capacity to cleave components of perineuronal nets [25, 26]. Perineuronal net degradation not only enhances interneuron vulnerability to environmental toxicity but also increases the plasticity and synapse formation of these cells [91]. It has been proposed that the initial loss of perineuronal nets in neurodegenerative diseases acts as an endogenous compensatory mechanism to facilitate synaptic plasticity and reduce cognitive decline [92]. However, with extended neuropathological progression of such diseases, further perineuronal net degradation is likely to accelerate degeneration and exacerbate dementia, thereby leading to advanced cognitive decline [92].

Our results show the presence of inhibitory postsynaptic densities of very immature 1-week-old dentate granule cells and their maturation time course. Moreover, they point to an in vivo role of GSK-3β in regulating GABAergic neurotransmission in the dentate gyrus. The functional alterations observed in GSK-3β-OE newborn dentate granule cells might be related to the observed impaired pattern separation capacity. In this regard, impairments in the multi-directional interactions involving immature (and mature) dentate granule cells, interneurons, and microglia [46] caused by the overexpression of this kinase have been shown to cause dramatic negative effects on memory formation dynamics. These functional impairments might be clinically relevant in the context of numerous neurological diseases in which the activity of this kinase is dysregulated.

## Supplementary Information

Below is the link to the electronic supplementary material.

Supplementary Figure S1. Visualization of Gephyrin^+^ clusters in newborn dentate granule cells (DGCs) of wild-type (WT) mice. A: Schematic diagram of a Gephyrin:GFP-encoding retrovirus, which was used to visualize inhibitory synapses made onto newborn dentate granule cells. B: Experimental design. C: Graphic scheme that illustrates the steps encompassed by the quantification of Parvalbumin (PV)^+^ volume surrounding Gephyrin^+^ clusters analysis. D: Colocalization between Gephyrin^+^ clusters and staining with an anti-Gephyrin antibody in newborn dentate granule cells transduced with a Gephyrin:GFP-encoding retrovirus. E: Absence of colocalization between PSD95 and Gephyrin in a newborn dentate granule cell transduced by retroviruses encoding PSD95:GFP and Gephyrin:mCherry. F: Newborn dentate granule cell Gephyrin^+^ postsynaptic clusters are surrounded by Bassoon^+^ presynaptic terminals. G – J: Percentage of the soma (G), its apical (H) and basal (I) domains, and axon (J) occupied by Gephyrin^+^ clusters in newborn dentate granule cells of distinct ages (1, 2, 3, 4 and 8 weeks post-infection). K – O: PV^+^ volume surrounding Gephyrin^+^ clusters in 1st order dendrites (K), soma (L) and its apical (M) and basal (N) domains, and axon (O) of newborn dentate granule cells of distinct ages. In D, E and F, Z-projection images, together with orthogonal views, are shown. In G – O, a nonparametric Kruskal-Wallis test, followed by a Dunn post hoc test, was used. In G – I, a minimum of 30 somas (G), and their apical (H) and basal (I) domains, of newborn dentate granule cells of each cell age, obtained from 4-5 mice, were analyzed. In J, a minimum of 30 axonal segments per cell age, obtained from 4-5 mice, were analyzed. In K – O, a minimum of 30 Gephyrin^+^ clusters of 1^st^ order dendrites (K), soma (L), apical (M), and basal (N) domains of the former, and axon (O) of newborn dentate granule cells of each age, obtained from 4-5 animals, were analyzed. Graphs represent mean values ± SEM. ML: Molecular layer. GCL: Granule cell layer. Magenta scale bar: 25 μm. Orange scale bar: 5 μm. Red scale bar: 2 µm. Light blue scale bar: 1 µm. Magenta triangles: Gephyrin^+^ clusters. * 0.05 > *p* ≥ 0.01; ** 0.01 > *p* ≥ 0.001; and *** 0.001 > *p* ≥ 0.0001

Supplementary Figure S2. Characterization of the inhibitory synapses made onto newborn dentate granule cells at distinct stages of maturation in wild-type (WT) mice. A: Serial segmented electron microscopy images of an inhibitory synapse made onto the dendrite of a WT 4-week-old dentate granule cell. B: Total density (number/µm^3^) of synaptic vesicles at inhibitory synapses made onto dentate granule cells of distinct ages (4, 8, and 60 weeks post-infection) in WT mice. C-E: Density (number/µm^3^) of docked (C), proximal (D), and distant (E) synaptic vesicles at inhibitory synapses made onto dentate granule cells of distinct ages (4, 8, and 60 weeks post-infection) in WT mice. In B – E, a nonparametric Kruskal-Wallis test, followed by a Dunn´s post hoc test was used. Between two and seven inhibitory synapses made onto dentate granule cells of each cell age were analyzed. Graphs represent mean values ± SEM. Yellow scale bar: 500 nm

Supplementary Figure S3. Inhibitory innervation of newborn dentate granule cells (DGCs) in GSK-3β-overexpressing (OE) mice. A – B: Density (number/µm) of Gephyrin^+^ clusters in the soma (A) and axon (B) of newborn dentate granule cells of distinct ages (1, 2, 3, 4 and 8 weeks post-infection) in wild-type (WT) and GSK-3β-OE mice. C – F: Area of Gephyrin^+^ clusters in 1^st^ (C) and higher (D) branching order dendrites, soma (E), and axon (F) of newborn dentate granule cells of distinct ages. G – H: Parvalbumin (PV)^+^ volume surrounding Gephyrin^+^ clusters in the 1^st^ order dendrites (G) and axon (H) of newborn dentate granule cells of distinct ages. I: Density of PV^+^ interneurons. J – K: Classification of dentate gyrus PV^+^ interneurons according to the intensity of Lectin^+^ (J) and Aggrecan^+^ (K) perineuronal nets (PNNs). L: Schematic diagram illustrating patch-clamp whole-cell recordings showing 1-week-old newborn dentate granule cells in red and embryonic dentate granule cells in purple. M – N: Representative miniature inhibitory postsynaptic currents (mIPSCs) traces recorded from embryonic (M) and 1-week-old newborn (N) dentate granule cells of GSK-3β- OE mice. O – Q: Frequency (O), amplitude (P), and slope (Q) of mIPSCs in developmentally generated and 1-week-old newborn dentate granule cells of WT and GSK-3β- OE mice. In A – H, a two-way ANOVA, followed by a Tukey post hoc test, was applied. In I, a Student t-test was used. A two-way ANOVA, followed by a Bonferroni post hoc test, was applied in J – K and O – Q. At least 30 somas (in A and E) and 30 axonal segments (in B and F) of newborn dentate granule cells of each age, obtained from 4-5 animals per genotype, were analyzed. In O – Q, six developmentally generated (embryonic) and six 1-week-old dentate granule cells obtained from four GSK-3β-OE mice, and seven developmentally generated (embryonic) and 10 1-week-old dentate granule cells obtained from four WT mice, were analyzed. Graphs represent mean values ± SEM. Vertical deflections represent mIPSCs. ^+^ 0.09 > *p* ≥ 0.05; * 0.05 > *p *≥ 0.01; ** 0.01 > *p* ≥ 0.001; and *** 0.001 > *p* ≥ 0.0001

Supplementary Figure S4. Morphology of newborn dentate granule cells in GSK-3β-overexpressing (OE) mice. A – J: Sholl´s analysis (A, C, E, G, and I) and total dendritic length (B, D, F, H, and J) of newborn dentate granule cells of distinct ages (1 (A – B), 2 (C – D), 3 (E – F), 4 (G – H) and 8 (I – J) weeks post-infection) in wild-type (WT) and GSK-3β-OE mice. In A, C, E, G, and I, a repeated measures ANOVA was used. In B, F, H, and J, a Student t-test was applied. In D, a Mann-Whitney U test was used. In A – J, 30 adult-born dentate granule cells of each age, obtained from 4-5 animals per genotype, were analyzed. Graphs represent mean values ± SEM. ^+^ 0.09 > *p* ≥ 0.05; * 0.05 > *p* ≥ 0.01; ** 0.01 > *p* ≥ 0.001; and *** 0.001 > *p* ≥ 0.0001

Supplementary Figure S5. Characterization of the perineuronal nets (PNNs) surrounding Parvalbumin (PV)^+^ interneurons located in distinct regions of the dentate gyrus in wild-type (WT) and GSK-3β-overexpressing (OE) mice. A - C: Density of PV^+^ interneurons in the Granule cell layer (GCL) (A), Subgranular zone (SGZ) (B) and Hilus (C) in WT and GSK-3β-OE mice. D – I: Lectin fluorescence intensity in PNNs that surround PV^+^ interneurons (D, F, and H) and classification of PV^+^ interneurons according to the intensity of Lectin^+^ PNNs (E, G, and I) in the GCL (D, E), SGZ (F, G) and Hilus (H, I). J - O: Aggrecan fluorescence intensity in PNNs that surround PV^+^ interneurons (J, L, and N) and classification of PV^+^ interneurons according to the intensity of Aggrecan^+^ PNNs (K, M, and O) in the GCL (J, K), SGZ (L, M) and Hilus (N, O). In A – C, D, H and J, a Student t-test was used. In F, L, and N, a nonparametric Mann-Whitney U test was applied. A two-way ANOVA, followed by a Bonferroni post hoc test, was used to analyze the data shown in E, G, I, K, M, and O. In A – O, 40-50 stacks of dentate gyrus images, obtained from 8-10 animals of each genotype, were analyzed. Graphs represent mean values ± SEM. * 0.05 > *p* ≥ 0.01; ** 0.01 > *p* ≥ 0.001; and *** 0.001 > *p* ≥ 0.000

Supplementary Table T1. List of primary antibodies used. The antigen, company, host species, catalog number, RRID, and concentration are shown

Supplementary Table T2. List of secondary antibodies used. The antibody, company, host species, catalog number, RRID, and concentration are shown

Supplementary file7. The results of all statistical comparisons included in this manuscript are presented. Each tab includes all the statistical comparisons that refer to a single Figure or Supplementary Figure

## Data Availability

The datasets generated in this study are available from the corresponding author upon reasonable request.
