# Supplementary figures and images for: GSK-3β orchestrates the inhibitory innervation of adult-born dentate granule cells in vivo
Source: Cell Mol Life Sci. 2023 Jul 23;80(8):225. doi: 10.1007/s00018-023-04874-w (PMC10363517; doi:10.1007/s00018-023-04874-w)

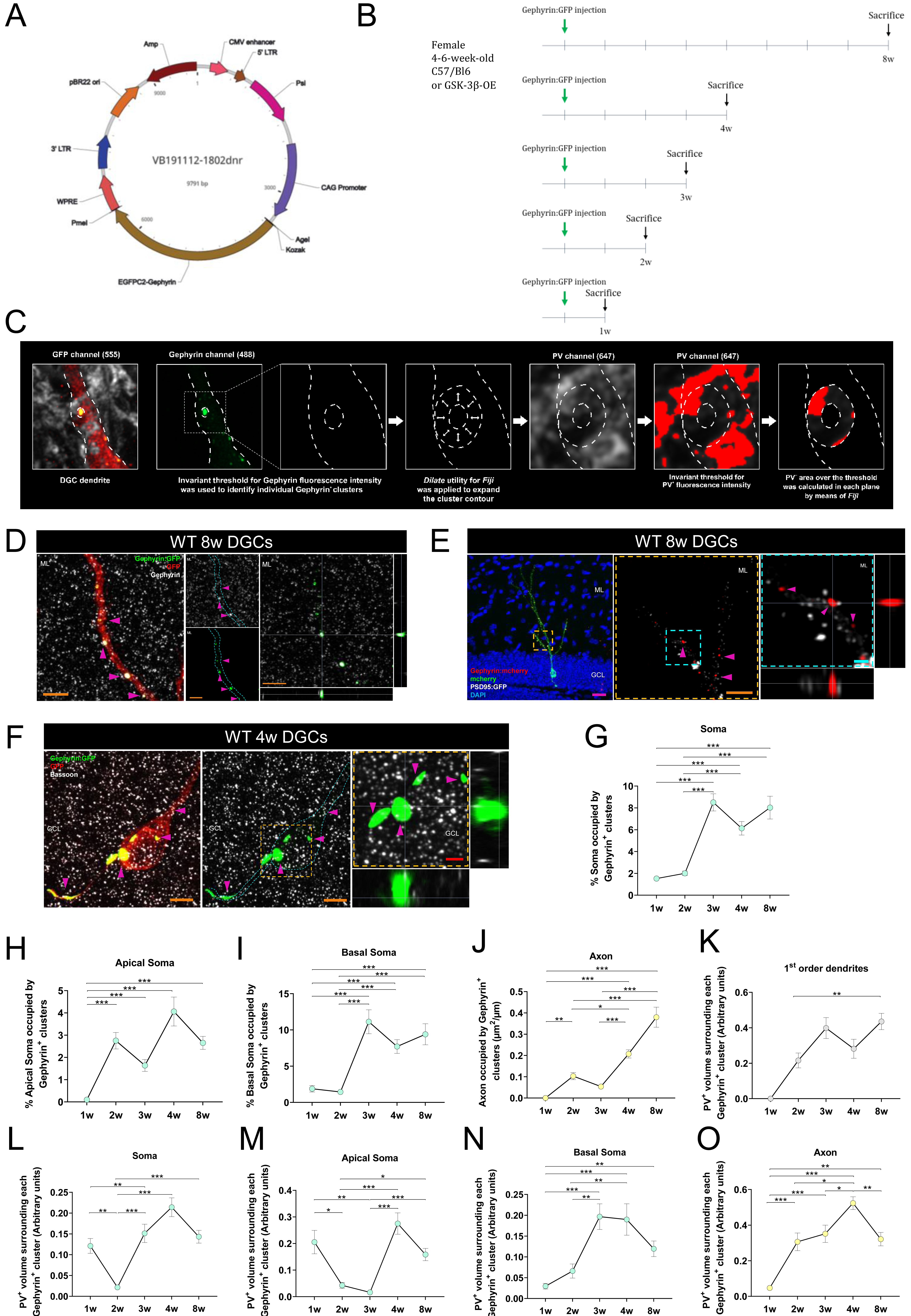

Supplement: Supplementary file 1 — Supplementary Figure S1. Visualization of Gephyrin+ clusters in newborn dentate granule cells (DGCs) of wild-type (WT) mice. A: Schematic diagram of a Gephyrin:GFP-encoding retrovirus, which was used to visualize inhibitory synapses made onto newborn dentate granule cells. B: Experimental design. C: Graphic scheme that illustrates the steps encompassed by the quantification of Parvalbumin (PV)+ volume surrounding Gephyrin+ clusters analysis. D: Colocalization between Gephyrin+ clusters and staining with an anti-Gephyrin antibody in newborn dentate granule cells transduced with a Gephyrin:GFP-encoding retrovirus. E: Absence of colocalization between PSD95 and Gephyrin in a newborn dentate granule cell transduced by retroviruses encoding PSD95:GFP and Gephyrin:mCherry. F: Newborn dentate granule cell Gephyrin+ postsynaptic clusters are surrounded by Bassoon+ presynaptic terminals. G – J: Percentage of the soma (G), its apical (H) and basal (I) domains, and axon (J) occupied by Gephyrin+ clusters in newborn dentate granule cells of distinct ages (1, 2, 3, 4 and 8 weeks post-infection). K – O: PV+ volume surrounding Gephyrin+ clusters in 1st order dendrites (K), soma (L) and its apical (M) and basal (N) domains, and axon (O) of newborn dentate granule cells of distinct ages. In D, E and F, Z-projection images, together with orthogonal views, are shown. In G – O, a nonparametric Kruskal-Wallis test, followed by a Dunn post hoc test, was used. In G – I, a minimum of 30 somas (G), and their apical (H) and basal (I) domains, of newborn dentate granule cells of each cell age, obtained from 4-5 mice, were analyzed. In J, a minimum of 30 axonal segments per cell age, obtained from 4-5 mice, were analyzed. In K – O, a minimum of 30 Gephyrin+ clusters of 1st order dendrites (K), soma (L), apical (M), and basal (N) domains of the former, and axon (O) of newborn dentate granule cells of each age, obtained from 4-5 animals, were analyzed. Graphs represent mean values ± SEM. ML: Mol [file 18_2023_4874_MOESM1_ESM.tif]

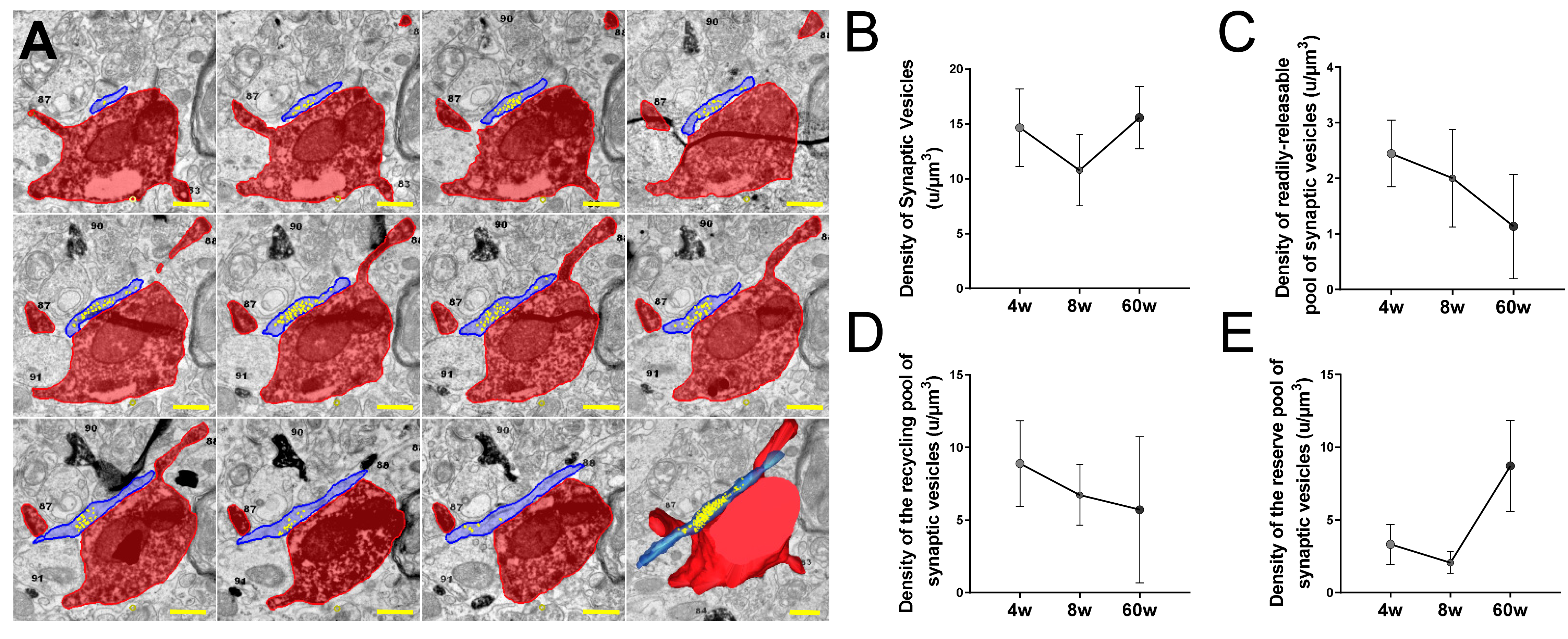

Supplement: Supplementary file 2 — Supplementary Figure S2. Characterization of the inhibitory synapses made onto newborn dentate granule cells at distinct stages of maturation in wild-type (WT) mice. A: Serial segmented electron microscopy images of an inhibitory synapse made onto the dendrite of a WT 4-week-old dentate granule cell. B: Total density (number/µm3) of synaptic vesicles at inhibitory synapses made onto dentate granule cells of distinct ages (4, 8, and 60 weeks post-infection) in WT mice. C-E: Density (number/µm3) of docked (C), proximal (D), and distant (E) synaptic vesicles at inhibitory synapses made onto dentate granule cells of distinct ages (4, 8, and 60 weeks post-infection) in WT mice. In B – E, a nonparametric Kruskal-Wallis test, followed by a Dunn´s post hoc test was used. Between two and seven inhibitory synapses made onto dentate granule cells of each cell age were analyzed. Graphs represent mean values ± SEM. Yellow scale bar: 500 nm [file 18_2023_4874_MOESM2_ESM.tif]

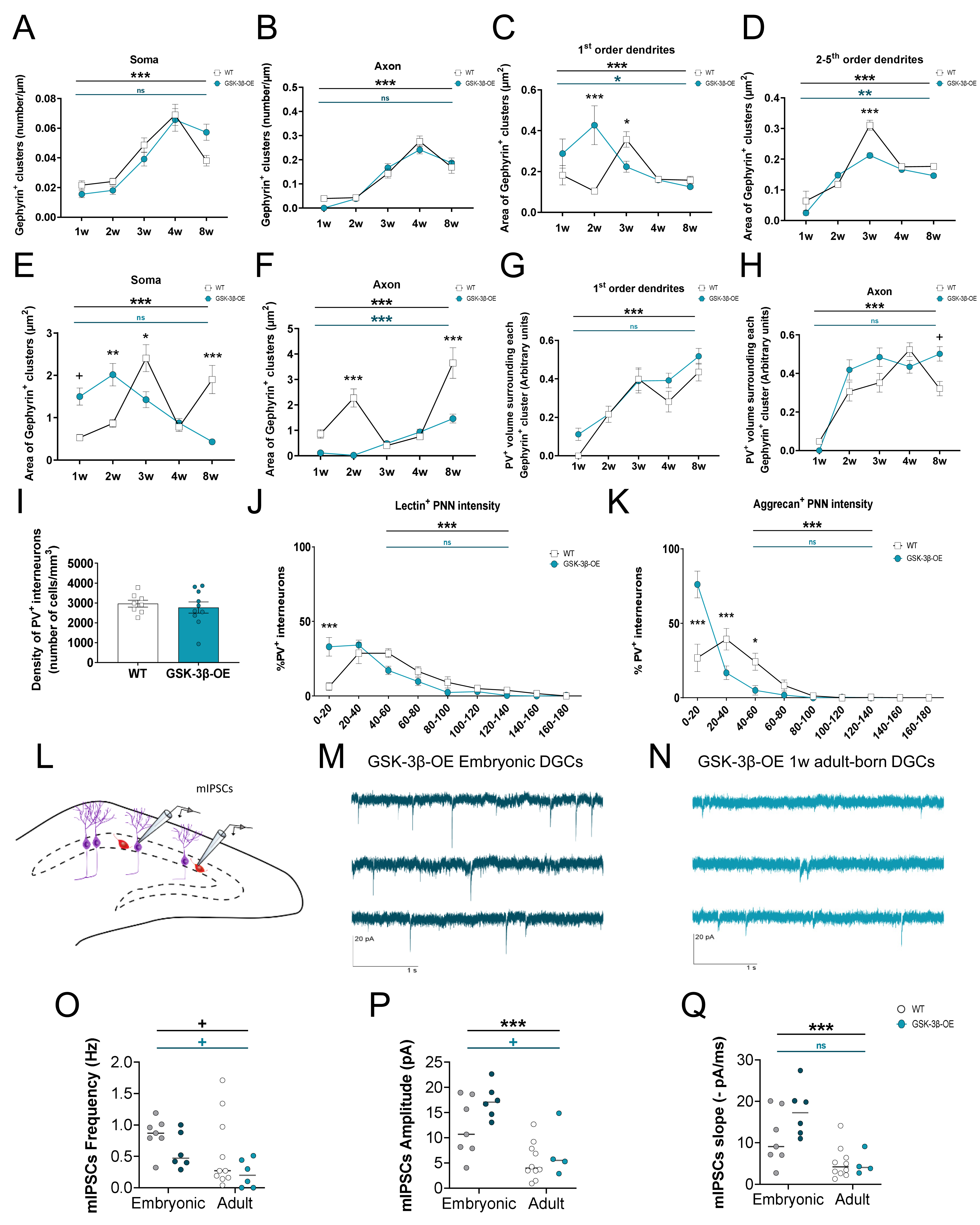

Supplement: Supplementary file 3 — Supplementary Figure S3. Inhibitory innervation of newborn dentate granule cells (DGCs) in GSK-3β-overexpressing (OE) mice. A – B: Density (number/µm) of Gephyrin+ clusters in the soma (A) and axon (B) of newborn dentate granule cells of distinct ages (1, 2, 3, 4 and 8 weeks post-infection) in wild-type (WT) and GSK-3β-OE mice. C – F: Area of Gephyrin+ clusters in 1st (C) and higher (D) branching order dendrites, soma (E), and axon (F) of newborn dentate granule cells of distinct ages. G – H: Parvalbumin (PV)+ volume surrounding Gephyrin+ clusters in the 1st order dendrites (G) and axon (H) of newborn dentate granule cells of distinct ages. I: Density of PV+ interneurons. J – K: Classification of dentate gyrus PV+ interneurons according to the intensity of Lectin+ (J) and Aggrecan+ (K) perineuronal nets (PNNs). L: Schematic diagram illustrating patch-clamp whole-cell recordings showing 1-week-old newborn dentate granule cells in red and embryonic dentate granule cells in purple. M – N: Representative miniature inhibitory postsynaptic currents (mIPSCs) traces recorded from embryonic (M) and 1-week-old newborn (N) dentate granule cells of GSK-3β- OE mice. O – Q: Frequency (O), amplitude (P), and slope (Q) of mIPSCs in developmentally generated and 1-week-old newborn dentate granule cells of WT and GSK-3β- OE mice. In A – H, a two-way ANOVA, followed by a Tukey post hoc test, was applied. In I, a Student t-test was used. A two-way ANOVA, followed by a Bonferroni post hoc test, was applied in J – K and O – Q. At least 30 somas (in A and E) and 30 axonal segments (in B and F) of newborn dentate granule cells of each age, obtained from 4-5 animals per genotype, were analyzed. In O – Q, six developmentally generated (embryonic) and six 1-week-old dentate granule cells obtained from four GSK-3β-OE mice, and seven developmentally generated (embryonic) and 10 1-week-old dentate granule cells obtained from four WT mice, were analyzed. Graphs represent mean values ± SEM. Vertic [file 18_2023_4874_MOESM3_ESM.tif]

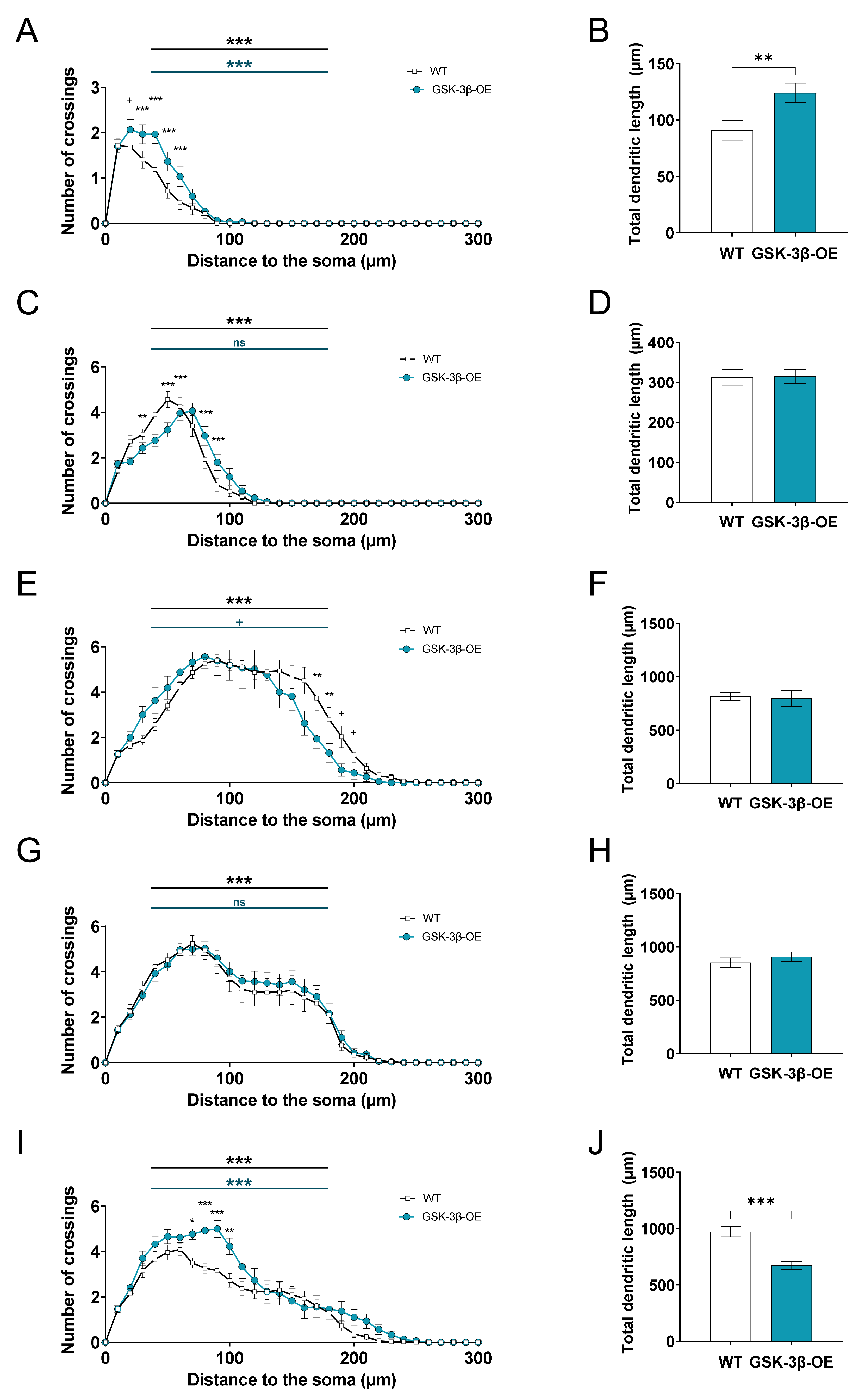

Supplement: Supplementary file 4 — Supplementary Figure S4. Morphology of newborn dentate granule cells in GSK-3β-overexpressing (OE) mice. A – J: Sholl´s analysis (A, C, E, G, and I) and total dendritic length (B, D, F, H, and J) of newborn dentate granule cells of distinct ages (1 (A – B), 2 (C – D), 3 (E – F), 4 (G – H) and 8 (I – J) weeks post-infection) in wild-type (WT) and GSK-3β-OE mice. In A, C, E, G, and I, a repeated measures ANOVA was used. In B, F, H, and J, a Student t-test was applied. In D, a Mann-Whitney U test was used. In A – J, 30 adult-born dentate granule cells of each age, obtained from 4-5 animals per genotype, were analyzed. Graphs represent mean values ± SEM. + 0.09 > p ≥ 0.05; * 0.05 > p ≥ 0.01; ** 0.01 > p ≥ 0.001; and *** 0.001 > p ≥ 0.0001 [file 18_2023_4874_MOESM4_ESM.tif]

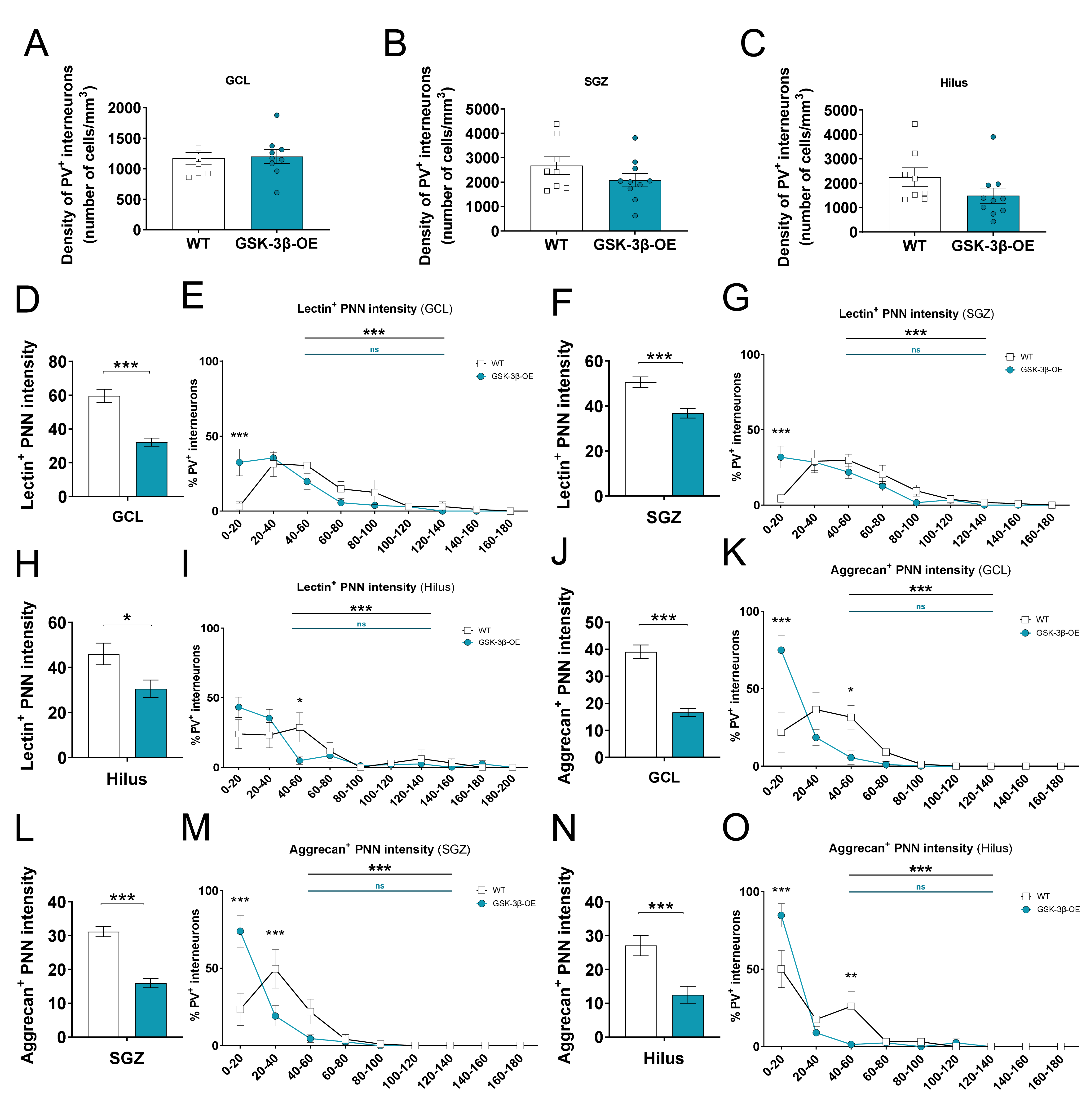

Supplement: Supplementary file 5 — Supplementary Figure S5. Characterization of the perineuronal nets (PNNs) surrounding Parvalbumin (PV)+ interneurons located in distinct regions of the dentate gyrus in wild-type (WT) and GSK-3β-overexpressing (OE) mice. A - C: Density of PV+ interneurons in the Granule cell layer (GCL) (A), Subgranular zone (SGZ) (B) and Hilus (C) in WT and GSK-3β-OE mice. D – I: Lectin fluorescence intensity in PNNs that surround PV+ interneurons (D, F, and H) and classification of PV+ interneurons according to the intensity of Lectin+ PNNs (E, G, and I) in the GCL (D, E), SGZ (F, G) and Hilus (H, I). J - O: Aggrecan fluorescence intensity in PNNs that surround PV+ interneurons (J, L, and N) and classification of PV+ interneurons according to the intensity of Aggrecan+ PNNs (K, M, and O) in the GCL (J, K), SGZ (L, M) and Hilus (N, O). In A – C, D, H and J, a Student t-test was used. In F, L, and N, a nonparametric Mann-Whitney U test was applied. A two-way ANOVA, followed by a Bonferroni post hoc test, was used to analyze the data shown in E, G, I, K, M, and O. In A – O, 40-50 stacks of dentate gyrus images, obtained from 8-10 animals of each genotype, were analyzed. Graphs represent mean values ± SEM. * 0.05 > p ≥ 0.01; ** 0.01 > p ≥ 0.001; and *** 0.001 > p ≥ 0.000 [file 18_2023_4874_MOESM5_ESM.tif]
