# Supplementary material for: GSK-3β orchestrates the inhibitory innervation of adult-born dentate granule cells in vivo
Source: Cell Mol Life Sci. 2023 Jul 23;80(8):225. doi: 10.1007/s00018-023-04874-w (PMC10363517; doi:10.1007/s00018-023-04874-w)
Supplement: Supplementary file 6 — Supplementary Table T1. List of primary antibodies used. The antigen, company, host species, catalog number, RRID, and concentration are shown [file 18_2023_4874_MOESM6_ESM.docx]

| ANTIGEN | COMPANY | HOST SPECIES | CATALOG NUMBER | RRID | CONCENTRATION |
| --- | --- | --- | --- | --- | --- |
| Aggrecan | MILLIPORE | RABBIT | AB1031 | RRID: AB_90460 | 1:500 |
| Ankyrin G | NEUROMAB | MOUSE | N106/36 | RRID: AB_2877524 | 1:1,000 |
| Basson | SYNAPTIC SYSTEMS | RABBIT | 141 003 | RRID: AB_887697 | 1:1,000 |
| Gephyrin | SYNAPTIC SYSTEMS | RABBIT | 147 018 | RRID: [AB_2651176](http://antibodyregistry.org/AB_2651176) | 1:500 |
| GFP | ABCAM | CHICKEN | AB13970 | RRID: AB_300798 | 1:500 |
| GFP | THERMO FISHER SCIENTIFIC | RABBIT | A-11122 | RRID: AB_221569 | 1:1,000 |
| mcherry | THERMO FISHER SCIENTIFIC | RAT | M11217 | RRID: AB_2536611 | 1:2,500 |
| Parvalbumin | SYNAPTIC SYSTEMS | MOUSE | 195 011 | RRID: AB_2619882 | 1:500 |
| Biotinylated Wisteria floribunda agglutinin  (WFA) - Lectin | SIGMA | - | L-1516 | RRID: AB_2620171 | 1:1,000 |
