# Supplementary material for: GSK-3β orchestrates the inhibitory innervation of adult-born dentate granule cells in vivo
Source: Cell Mol Life Sci. 2023 Jul 23;80(8):225. doi: 10.1007/s00018-023-04874-w (PMC10363517; doi:10.1007/s00018-023-04874-w)
Supplement: Supplementary file 7 — Supplementary Table T2. List of secondary antibodies used. The antibody, company, host species, catalog number, RRID, and concentration are shown [file 18_2023_4874_MOESM7_ESM.docx]

| ANTIBODY | COMPANY | HOST SPECIES | CATALOG NUMBER | RRID | CONCENTRATION |
| --- | --- | --- | --- | --- | --- |
| Alexa-488 anti-chicken | THERMO FISHER SCIENTIFIC | GOAT | A-11039 | RRID: AB_142924 | 1:1,000 |
| Alexa-488 anti-mouse | THERMO FISHER SCIENTIFIC | DONKEY | A-21202 | RRID: AB_141607 | 1:1,000 |
| Alexa-555 anti-biotin | THERMO FISHER SCIENTIFIC | STREPTAVIDIN | S-32355 | RRID: AB_2571525 | 1:1,000 |
| Alexa-555 anti-chicken | THERMO FISHER SCIENTIFIC | GOAT | A-21437 | RRID: AB_2535858 | 1:1,000 |
| Alexa-555 anti-rabbit | THERMO FISHER SCIENTIFIC | DONKEY | A-31572 | RRID: AB_162543 | 1:1,000 |
| Alexa-555 anti-rat | THERMO FISHER SCIENTIFIC | GOAT | A-21434 | RRID: AB_141733 | 1:1,000 |
| Alexa-647 anti-mouse | THERMO FISHER SCIENTIFIC | DONKEY | A-31571 | RRID: AB_162542 | 1:1,000 |
| Alexa-647 anti-rabbit | THERMO FISHER SCIENTIFIC | DONKEY | A-31573 | RRID: AB_2536183 | 1:1,000 |
| Alexa-647 anti-rat | THERMO FISHER SCIENTIFIC | GOAT | A-21247 | RRID: AB_141778 | 1:1,000 |
