# Supplementary material for: GSK-3β orchestrates the inhibitory innervation of adult-born dentate granule cells in vivo
Source: Cell Mol Life Sci. 2023 Jul 23;80(8):225. doi: 10.1007/s00018-023-04874-w (PMC10363517; doi:10.1007/s00018-023-04874-w)
Supplement: Supplementary file 8 — Supplementary file7. The results of all statistical comparisons included in this manuscript are presented. Each tab includes all the statistical comparisons that refer to a single Figure or Supplementary Figure [file 18_2023_4874_MOESM8_ESM.pdf]

FIGURE 1:

Fig 1G	Density of Gephyrin⁺ clusters in 1st order dendrites of adult-born DGCs				
	KRUSKAL-WALLIS TEST				
	df		K	p	
	4 / 140		76.08	**<.001**	
	1w	2w	3w	4w	8w
1w	--	0.72	<.001	<.001	<.001
2w	>.999	--	<.001	<.001	0.003
3w	<.001	<.001	--	>.999	>.999
4w	<.001	<.001	>.999	--	0.383
8w	<.001	0.003	>.999	0.383	--

Fig 1G	Density of Gephyrin⁺ clusters in 2nd order dendrites of adult-born DGCs				
	KRUSKAL-WALLIS TEST				
	df		K	p	
	4 / 141		94.37	**<.001**	
	1w	2w	3w	4w	8w
1w	--	>.999	<.001	<.001	<.001
2w	>.999	--	<.001	<.001	<.001
3w	<.001	<.001	--	>.999	>.999
4w	<.001	<.001	>.999	--	>.999
8w	<.001	<.001	>.999	>.999	--

Fig 1G	Density of Gephyrin⁺ clusters in 3rd order dendrites of adult-born DGCs				
	KRUSKAL-WALLIS TEST				
	df		K	p	
	4 / 150		108.8	**<.001**	
	1w	2w	3w	4w	8w
1w	--	0.796	<.001	<.001	<.001
2w	0.796	--	<.001	<.001	<.001
3w	<.001	<.001	--	>.999	>.999
4w	<.001	<.001	>.999	--	>.999
8w	<.001	<.001	>.999	>.999	--

Fig 1G	Density of Gephyrin⁺ clusters in 4th order dendrites of adult-born DGCs			
	KRUSKAL-WALLIS TEST			
	df	K	p	
	3 / 123	63.73	**<.001**	
	2w	3w	4w	8w
2w	--	<.001	<.001	<.001
3w	<.001	--	0.018	0.039
4w	<.001	0.018	--	>.999
8w	<.001	0.039	>.999	--

Fig 1G	Density of Gephyrin⁺ clusters in 5th order dendrites of adult-born DGCs			
	KRUSKAL-WALLIS TEST			
	df	K	p	
	3 / 122	68.94	**<.001**	
	2w	3w	4w	8w
2w	--	<.001	<.001	<.001
3w	<.001	--	0.017	0.294
4w	<.001	0.017	--	>.999
8w	<.001	0.294	>.999	--

Fig 1H	Density of Gephyrin^{+} clusters in the soma of adult-born DGCs				
	KRUSKAL-WALLIS TEST				
	df		K	p	
	4 / 155		41.2	<.001	
	1w	2w	3w	4w	8w
1w	--	>.999	<.001	<.001	0.021
2w	>.999	--	0.002	<.001	0.129
3w	<.001	0.002	--	>.999	>.999
4w	<.001	<.001	>.999	--	0.426
8w	0.021	0.129	>.999	0.426	--

Fig 1I	Density of Gephyrin^{+} clusters in the axon of adult-born DGCs				
	KRUSKAL-WALLIS TEST				
	df		K	p	
	4 / 146		75.53	<.001	
	1w	2w	3w	4w	8w
1w	--	>.999	<.001	<.001	<.001
2w	>.999	--	0.001	<.001	<.001
3w	<.001	0.001	--	0.014	>.999
4w	<.001	<.001	0.014	--	0.084
8w	<.001	<.001	>.999	0.084	--

Fig 1J	Area of Gephyrin^{+} clusters in 1st order dendrites of adult-born DGCs				
	KRUSKAL-WALLIS TEST				
	df		K	p	
	4 / 85		27.25	<.001	
	1w	2w	3w	4w	8w
1w	--	>.999	0.512	>.999	>.999
2w	>.999	--	<.001	0.364	0.88
3w	0.512	<.001	--	0.005	0.01
4w	>.999	0.364	0.005	--	>.999
8w	>.999	0.88	0.01	>.999	--

Fig 1J	Area of Gephyrin^{+} clusters in 2nd order dendrites of adult-born DGCs				
	KRUSKAL-WALLIS TEST				
	df		F	p	
	4 / 95		35.95	<.001	
	1w	2w	3w	4w	8w
1w	--	>.999	0.033	>.999	0.503
2w	>.999	--	<.001	>.999	0.069
3w	0.033	<.001	--	<.001	0.347
4w	>.999	>.999	<.001	--	0.1
8w	0.503	0.069	0.347	0.1	--

Fig 1J	Area of Gephyrin^{+} clusters in 3rd order dendrites of adult-born DGCs				
	KRUSKAL-WALLIS TEST				
	df		F	p	
	4 / 104		38.46	<.001	
	1w	2w	3w	4w	8w
1w	--	>.999	0.115	0.974	>.999
2w	>.999	--	<.001	0.033	0.172
3w	0.115	<.001	--	0.008	<.001
4w	0.974	0.033	0.008	--	>.999
8w	>.999	0.172	<.001	>.999	--

Fig 1J	Area of Gephyrin$^+$ clusters in 4th order dendrites of adult-born DGCs			
	KRUSKAL-WALLIS TEST			
	df	K	p	
	3 / 113	55.79	**<.001**	
	2w	3w	4w	8w
2w	--	<.001	<.001	0.008
3w	<.001	--	<.001	<.001
4w	<.001	<.001	--	>.999
8w	0.008	<.001	>.999	--

Fig 1J	Area of Gephyrin$^+$ clusters in 5th order dendrites of adult-born DGCs			
	KRUSKAL-WALLIS TEST			
	df	K	p	
	3 / 106	45.76	**<.001**	
	2w	3w	4w	8w
2w	--	<.001	<.001	0.007
3w	<.001	--	0.368	0.001
4w	<.001	0.368	--	0.442
8w	0.007	0.001	0.442	--

Fig 1K	Area of Gephyrin$^+$ clusters in the soma of adult-born DGCs				
	KRUSKAL-WALLIS TEST				
	df		K	p	
	4 / 131		38.08	**<.001**	
	1w	2w	3w	4w	8w
1w	--	0.895	<.001	>.999	0.002
2w	0.895	--	<.001	>.999	0.282
3w	<.001	<.001	--	<.001	>.999
4w	>.999	>.999	<.001	--	0.134
8w	0.002	0.282	>.999	0.134	--

Fig 1L	Area of Gephyrin$^+$ clusters in the axon of adult-born DGCs				
	KRUSKAL-WALLIS TEST				
	df		K	p	
	4 / 123		28.55	**<.001**	
	1w	2w	3w	4w	8w
1w	--	0.997	0.886	>.999	0.056
2w	0.997	--	0.002	0.669	>.999
3w	0.886	0.002	--	0.378	<.001
4w	>.999	0.669	0.378	--	0.016
8w	0.056	>.999	<.001	0.016	--

FIGURE 2:

Fig 2A — Density of Gephyrin$^+$ clusters in 1st order dendrites of adult-born DGCs

Two-way ANOVA

		GSK-3-OE 1w	GSK-3-OE 2w	GSK-3-OE 3w	GSK-3-OE 4w	GSK-3-OE 8w
WT	1w	**>.999**	>.999	<.001	<.001	<.001
	2w	>.999	**>.999**	0.007	<.001	<.001
	3w	<.001	<.001	**0.628**	0.007	0.231
	4w	<.001	<.001	0.019	**0.377**	0.976
	8w	<.001	<.001	>.999	<.001	**0.006**

INTER-SUBJECT EFFECT

Effect	df	F	p
Age	4 / 292	59.230	<.001
Genotipe	1 / 292	4.01	0.046
Age*Genotipe	4 / 292	5.118	<.001

Fig 2B — Density of Gephyrin$^+$ clusters in 2-5th order dendrites of adult-born DGCs

Two-way ANOVA

		GSK-3-OE 1w	GSK-3-OE 2w	GSK-3-OE 3w	GSK-3-OE 4w	GSK-3-OE 8w
WT	1w	**>.999**	0.276	<.001	<.001	<.001
	2w	0.097	**0.999**	<.001	<.001	<.001
	3w	<.001	<.001	**0.026**	<.001	0.027
	4w	<.001	<.001	<.001	**>.999**	0.988
	8w	<.001	<.001	<.001	>.999	**0.982**

INTER-SUBJECT EFFECT

Effect	df	F	p
Age	4 / 1067	297.200	<.001
Genotipe	1 / 1067	4.932	0.027
Age*Genotipe	4 / 1067	1.674	0.154

Fig 2D — Colocalization between Basson and Gephyrin

Mann Whitney test

	U	p
WT vs GSK-3-OE	1144	0.004

Fig 2E — Percentage adult-born DGCs surrounded by PV$^+$ interneurons

Chi-square

	df	z	p
WT vs GSK-3-OE	1 / 13.13	3.623	<.001

Fig 2G — PV$^+$ area surrounding Gephyrin$^+$ clusters in the soma of adult-born DGCs

Two-way ANOVA

		GSK-3-OE 1w	GSK-3-OE 2w	GSK-3-OE 3w	GSK-3-OE 4w	GSK-3-OE 8w
WT	1w	**0.204**	<.001	0.228	0.656	0.009
	2w	>.999	**<.001**	<.001	<.001	<.001
	3w	0.015	<.001	**0.853**	0.996	0.198
	4w	<.001	0.085	>.999	**0.972**	>.999
	8w	0.018	<.001	0.621	0.963	**0.059**

INTER-SUBJECT EFFECT

Effect	df	F	p
Age	4 / 655	9.041	<.001
Genotipe	1 / 655	18.820	<.001
Age*Genotipe	4 / 655	18.590	<.001

Fig 2H — Intensity of Lectin in PNNs surrounding PV interneurons

Mann Whitney test

	U	p
WT vs GSK-3-OE	11	0.009

Fig 2I — Intensity of Aggrecan in PNNs surrounding PV interneurons

Student t- Test

	t	df	p
WT vs GSK-3-OE	5.021	15	<.001

FIGURE 3:

Fig 3E	Frequency of mIPSCs in developmentally generated and 8-week-old adult-born DGCs of WT and GSK-3β-OE mice	
	Two-way ANOVA	
	GSK-3-OE	
WT	Embryonic	Adult
Embryonic	>0.9999	--
Adult	--	>0.9999

INTER-SUBJECT EFFECT		
Cell age		
df	F	p
1 / 41	2.024	0.1624
Genotipe		
df	F	p
1 / 41	0.4897	0.488
Age*Genotipe		
df	F	p
1 / 41	0.0418	0.839

Fig 3F	Amplitude of mIPSCs in developmentally generated and 8-week-old adult-born DGCs of WT and GSK-3β-OE mice	
	Two-way ANOVA	
	GSK-3-OE	
WT	Embryonic	Adult
Embryonic	0.4023	--
Adult	--	0.0406

INTER-SUBJECT EFFECT		
Cell age		
df	F	p
1 / 41	0.091	0.7641
Genotipe		
df	F	p
1 / 41	6.683	0.0134
Age*Genotipe		
df	F	p
1 / 41	0.438	0.5119

Fig 3G	Slope of mIPSCs in developmentally generated and 8-week-old adult-born DGCs of WT and GSK-3β-OE mice	
	Two-way ANOVA	
	GSK-3-OE	
WT	Embryonic	Adult
Embryonic	0.7579	--
Adult	--	0.0221

INTER-SUBJECT EFFECT		
Cell age		
df	F	p
1 / 41	0.025	0.8748
Genotipe		
df	F	p
1 / 41	5.993	0.0187
Age*Genotipe		
df	F	p
1 / 41	1.280	0.2645

Fig 3H	AIS Length		
	Two-way ANOVA		
	GSK-3-OE		
WT	2w	4w	8w
2w	**0.022**	>.999	>.999
4w	<.001	**0.026**	0.005
8w	<.001	<.001	**<.001**

INTER-SUBJECT EFFECT		
Age		
df	F	p
2 / 166	17.940	<.001
Genotipe		
df	F	p
1 / 166	47.110	<.001
Age*Genotipe		
df	F	p
2 / 166	2.085	0.128

Fig 3I	AIS Starting point		
	Two-way ANOVA		
	GSK-3-OE		
WT	2w	4w	8w
2w	**0.373**	0.909	0.17
4w	0.073	**0.385**	0.016
8w	0.569	0.985	**0.33**

INTER-SUBJECT EFFECT		
Age		
df	F	p
2 / 167	1.940	0.147
Genotipe		
df	F	p
1 / 167	11.620	<.001
Age*Genotipe		
df	F	p
2 / 167	0.0268	0.974

Fig 3N	Colocalization between GFP and Syn in CA3 MFTs			INTER-SUBJECT EFFECT		
	Two-way ANOVA			Age		
				df	F	p
				2 / 98	27.960	<.001
WT	GSK-3-OE			Genotipe		
				df	F	p
	2w	4w	8w	1 / 98	3.688	0.058
2w	**>.999**	0.035	<.001	Age*Genotipe		
4w	0.845	**0.238**	<.001	df	F	p
8w	<.001	0.867	**0.832**	2 / 98	1.181	0.311

Fig 3O	Colocalization between GFP and Syn in CA2 MFTs			INTER-SUBJECT EFFECT		
	Two-way ANOVA			Age		
				df	F	p
				2 / 65	24.100	<.001
WT	GSK-3-OE			Genotipe		
				df	F	p
	2w	4w	8w	1 / 65	12.52	<.001
2w	**0.992**	<.001	<.001	Age*Genotipe		
4w	0.906	**0.008**	<.001	df	F	p
8w	0.001	0.956	**0.285**	2 / 65	1.999	0.144

Fig 3P	Total exploratory time (s) during the 3rd day of the NLP test		
	Student t- Test		
	t	df	p
WT vs GSK-3-OE	3.774	15	0.002

Fig 3Q	Memory index during the 3rd day of the NLP test		
	Student t- Test		
	t	df	p
WT vs GSK-3-OE	2.352	15	0.033

SUPPLEMENTARY FIGURE S1:

Fig S1G	Percentage of the soma occupied by Gephyrin$^+$ clusters in adult-born DGCs				
	KRUSKAL-WALLIS TEST				
	df	K	p		
	4 / 155	80.29	**<.001**		
	1w	2w	3w	4w	8w
**1w**	--	>.999	<.001	<.001	<.001
**2w**	>.999	--	<.001	<.001	<.001
**3w**	<.001	<.001	--	>.999	>.999
**4w**	<.001	<.001	>.999	--	>.999
**8w**	<.001	<.001	>.999	>.999	--

Fig S1H	Percentage of the Apical Soma occupied by Gephyrin$^+$ clusters in adult-born DGCs				
	KRUSKAL-WALLIS TEST				
	df	K	p		
	4 / 161	45.46	**<.001**		
	1w	2w	3w	4w	8w
**1w**	--	<.001	0.002	<.001	<.001
**2w**	<.001	--	>.999	>.999	>.999
**3w**	0.002	>.999	--	0.176	>.999
**4w**	<.001	>.999	0.176	--	>.999
**8w**	<.001	>.999	>.999	>.999	--

Fig S1I	Percentage of the Basal Soma occupied by Gephyrin$^+$ clusters in adult-born DGCs				
	KRUSKAL-WALLIS TEST				
	df	K	p		
	4 / 155	62.58	**<.001**		
	1w	2w	3w	4w	8w
**1w**	--	>.999	<.001	<.001	<.001
**2w**	>.999	--	<.001	<.001	<.001
**3w**	<.001	<.001	--	>.999	>.999
**4w**	<.001	<.001	>.999	--	>.999
**8w**	<.001	<.001	>.999	>.999	--

Fig S1J	Percentage of the axon occupied by Gephyrin$^+$ clusters in adult-born DGCs				
	KRUSKAL-WALLIS TEST				
	df	K	p		
	4 / 125	73.23	**<.001**		
	1w	2w	3w	4w	8w
**1w**	--	0.003	0.235	<.001	<.001
**2w**	0.003	--	>.999	0.024	<.001
**3w**	0.235	>.999	--	<.001	<.001
**4w**	<.001	0.024	<.001	--	>.999
**8w**	<.001	<.001	<.001	>.999	--

Fig S1K	PV+ area surrounding Gephyrin+ clusters in the 1st order dendrites of adult-born DGCs				
	KRUSKAL-WALLIS TEST				
	df	K	p		
	4 / 141	13.78	**0.008**		
	1w	2w	3w	4w	8w
**1w**	--	>.999	0.876	>.999	0.643
**2w**	>.999	--	0.309	>.999	0.03
**3w**	0.876	0.309	--	>.999	>.999
**4w**	>.999	>.999	>.999	--	0.236
**8w**	0.643	0.03	>.999	0.236	--

Fig S1L	PV+ area surrounding Gephyrin+ clusters in the Soma of adult-born DGCs				
	KRUSKAL-WALLIS TEST				
	df	K	p		
	4 / 141	52.26	**<.001**		
	1w	2w	3w	4w	8w
**1w**	--	0.002	>.999	0.005	>.999
**2w**	0.002	--	<.001	<.001	<.001
**3w**	>.999	<.001	--	0.113	>.999
**4w**	0.005	<.001	0.113	--	0.398
**8w**	>.999	<.001	>.999	0.398	--

Fig S1M	PV+ area surrounding Gephyrin+ clusters in the Apical Soma of adult-born DGCs				
	KRUSKAL-WALLIS TEST				
	df	K	p		
	4 / 135	42.34	**<.001**		
	1w	2w	3w	4w	8w
**1w**	--	0.022	0.001	>.999	>.999
**2w**	0.022	--	>.999	<.001	0.017
**3w**	0.001	>.999	--	<.001	<.001
**4w**	>.999	<.001	<.001	--	0.756
**8w**	>.999	0.017	<.001	0.756	--

Fig S1N	PV+ area surrounding Gephyrin+ clusters in the Basal Soma of adult-born DGCs				
	KRUSKAL-WALLIS TEST				
	df	K	p		
	4 / 156	37.26	**<.001**		
	1w	2w	3w	4w	8w
**1w**	--	>.999	<.001	<.001	0.007
**2w**	>.999	--	0.001	0.002	0.192
**3w**	<.001	0.001	--	>.999	0.909
**4w**	<.001	0.002	>.999	--	>.999
**8w**	0.007	0.192	0.909	>.999	--

Fig S1O	PV+ area surrounding Gephyrin+ clusters in the axon of adult-born DGCs				
	KRUSKAL-WALLIS TEST				
	df	K	p		
	4 / 223	41.49	**<.001**		
	1w	2w	3w	4w	8w
**1w**	--	<.001	<.001	<.001	0.002
**2w**	<.001	--	>.999	0.025	>.999
**3w**	<.001	>.999	--	0.032	>.999
**4w**	<.001	0.025	0.032	--	0.004
**8w**	0.002	>.999	>.999	0.004	--

SUPPLEMENTARY FIGURE S2:

Fig S2B	Total density of synaptic vesicles in inhibitory synapses made onto adult-born DGCs		
	KRUSKAL-WALLIS TEST		
	df	K	p
	2 / 13	0.7465	0.721
	4w	8w	60w
4w	--	>.999	>.999
8w	>.999	--	>.999
60w	>.999	>.999	--

Fig S2C	Density of the readily-releasable pool of synaptic vesicles in inhibitory synapses made onto adult-born DGCs		
	KRUSKAL-WALLIS TEST		
	df	K	p
	2 / 13	1.137	0.606
	4w	8w	60w
4w	--	>.999	>.999
8w	>.999	--	>.999
60w	>.999	>.999	--

Fig S2D	Density of the recycling pool of synaptic vesicles in inhibitory synapses made onto adult-born DGCs		
	KRUSKAL-WALLIS TEST		
	df	K	p
	2 / 13	0.6782	0.74
	4w	8w	60w
4w	--	>.999	>.999
8w	>.999	--	>.999
60w	>.999	>.999	--

Fig S2E	Density of the reserve pool of synaptic vesicles in inhibitory synapses made onto adult-born DGCs		
	KRUSKAL-WALLIS TEST		
	df	K	p
	2 / 13	3.233	0.213
	4w	8w	60w
4w	--	>.999	0.313
8w	>.999	--	0.265
60w	0.313	0.265	--

SUPPLEMENTARY FIGURE S3:

Fig S3A		Density of Gephyrin$^+$ clusters in the Soma of adult-born DGCs					INTER-SUBJECT EFFECT		
		Two-way ANOVA					Age		
							df	F	p
		GSK-3-OE					4 / 306	39.010	<.001
		1w	2w	3w	4w	8w	Genotipe		
WT	1w	**0.995**	>.999	0.174	<.001	<.001	df	F	p
	2w	0.95	**0.993**	0.356	<.001	<.001	1 / 306	0.156	0.693
	3w	<.001	<.001	**0.917**	0.242	0.952	Age*Genotipe		
	4w	<.001	<.001	<.001	**>.999**	0.756	df	F	p
	8w	0.026	0.045	>.999	0.002	**0.114**	4 / 306	3.061	0.017

Fig S3B		Density of Gephyrin$^+$ clusters in the Axon of adult-born DGCs					INTER-SUBJECT EFFECT		
		Two-way ANOVA					Age		
							df	F	p
		GSK-3-OE					4 / 249	51.990	<.001
		1w	2w	3w	4w	8w	Genotipe		
WT	1w	**0.894**	>.999	<.001	<.001	<.001	df	F	p
	2w	0.933	**>.999**	0.005	<.001	<.001	1 / 249	0.164	0.686
	3w	<.001	0.131	**0.997**	0.005	0.808	Age*Genotipe		
	4w	<.001	<.001	<.001	**0.914**	0.012	df	F	p
	8w	<.001	0.015	>.999	0.105	**>.999**	4 / 249	1.353	0.251

Fig S3C		Area of Gephyrin$^+$ clusters in 1st order dendrites of adult-born DGCs					INTER-SUBJECT EFFECT		
		Two-way ANOVA					Age		
							df	F	p
		GSK-3-OE					4 / 187	8.961	<.001
		1w	2w	3w	4w	8w	Genotipe		
WT	1w	**0.914**	0.027	>.999	>.999	0.998	df	F	p
	2w	0.115	**<.001**	0.445	0.988	>.999	1 / 187	4.730	0.031
	3w	0.946	0.919	**0.031**	<.001	<.001	Age*Genotipe		
	4w	0.325	<.001	0.87	**>.999**	0.997	df	F	p
	8w	0.405	<.001	0.915	>.999	**>.999**	4 / 187	10.440	<.001

Fig S3D		Area of Gephyrin$^+$ clusters in 2-5th order dendrites of adult-born DGCs					INTER-SUBJECT EFFECT		
		Two-way ANOVA					Age		
							df	F	p
		GSK-3-OE					4 / 855	74.1	<.001
		1w	2w	3w	4w	8w	Genotipe		
WT	1w	**0.997**	0.965	<.001	0.21	648	df	F	p
	2w	<.001	**0.611**	<.001	0.005	0.458	1 / 855	8.41	0.04
	3w	<.001	<.001	**<.001**	<.001	<.001	Age*Genotipe		
	4w	<.001	0.556	0.034	**0.998**	0.186	df	F	p
	8w	<.001	0.527	0.052	0.996	**0.172**	4 / 855	14.13	<.001

Fig S3E		Area of Gephyrin$^+$ clusters in the soma of adult-born DGCs					INTER-SUBJECT EFFECT		
		Two-way ANOVA					Age		
							df	F	p
		GSK-3-OE					4 / 259	8.533	<.001
		1w	2w	3w	4w	8w	Genotipe		
WT	1w	**0.062**	<.001	0.062	0.971	>.999	df	F	p
	2w	0.552	**0.002**	0.611	>.999	0.934	1 / 259	0.117	0.732
	3w	0.094	0.923	**0.02**	<.001	<.001	Age*Genotipe		
	4w	0.432	0.001	0.484	**>.999**	0.987	df	F	p
	8w	0.96	>.999	0.838	0.022	**<.001**	4 / 259	14.930	<.001

Fig S3F		Area of Gephyrin$^+$ clusters in the axon of adult-born DGCs					INTER-SUBJECT EFFECT		
		Two-way ANOVA					Age		
							df	F	p
		GSK-3-OE					4 / 216	17.350	<.001
		1w	2w	3w	4w	8w	Genotipe		
WT	1w	**0.983**	0.773	0.998	>.999	0.945	df	F	p
	2w	0.023	**<.001**	<.001	0.01	0.521	1 / 216	22.240	<.001
	3w	>.999	0.998	**>.999**	0.95	0.281	Age*Genotipe		
	4w	0.991	0.773	>.999	**>.999**	0.714	df	F	p
	8w	<.001	<.001	<.001	<.001	**<.001**	4 / 216	8.831	<.001

Fig S3G		PV^{+} area surrounding Gephyrin^{+} clusters in the 1st order dendrites of newborn DGCs					INTER-SUBJECT EFFECT		
		Two-way ANOVA					Age		
							df	F	p
		GSK-3-OE					4 / 312	9.093	<.001
		1w	2w	3w	4w	8w	Genotipe		
WT	1w	**>.999**	>.999	0.947	0.942	0.751	df	F	p
	2w	0.983	**>.999**	0.365	0.155	<.001	1 / 312	0.802	0.371
	3w	0.067	0.276	**>.999**	>.999	0.751	Age*Genotipe		
	4w	0.729	0.997	0.919	**0.801**	0.022	df	F	p
	8w	0.009	0.034	>.999	0.999	**0.93**	4 / 312	0.573	0.682

Fig S3H		PV^{+} area surrounding Gephyrin^{+} clusters in the axon of newborn DGCs					INTER-SUBJECT EFFECT		
		Two-way ANOVA					Age		
							df	F	p
		GSK-3-OE					4 / 388	9.552	<.001
		1w	2w	3w	4w	8w	Genotipe		
WT	1w	**>.999**	0.001	<.001	<.001	<.001	df	F	p
	2w	0.415	**0.869**	0.161	0.517	0.089	1 / 388	2.367	0.125
	3w	0.2	0.993	**0.44**	0.887	0.281	Age*Genotipe		
	4w	0.003	0.815	>.999	**0.729**	>.999	df	F	p
	8w	0.302	0.889	0.11	0.451	**0.053**	4 / 388	4.048	0.003

Fig S3I	Density of PV^{+} interneurons		
	Student t- Test		
	t	df	p
WT vs GSK-3-OE	0.5512	16	0.589

Fig S3J		Classification of DG Parvalbumin (PV)$^{+}$ interneurons according to the intensity of Lectin^{+} perineuronal nets (PNNs)											
		Two-way ANOVA									INTER-SUBJECT EFFECT		
		GSK-3-OE									Intensity		
		0-20	20-40	40-60	60-80	80-100	100-120	120-140	140-160	160-180	df	F	p
WT	0-20	**<.001**	<.001	0.344	>.999	>.999	>.999	0.996	0.99	0.996	11 / 192	34.570	<.001
	20-40	>.999	**0.999**	0.22	<.001	<.001	<.001	<.001	<.001	<.001			
	40-60	>.999	0.998	**0.231**	<.001	<.001	<.001	<.001	<.001	<.001	Genotipe		
	60-80	0.003	<.001	>.999	**0.975**	0.031	0.051	0.004	0.003	0.004	df	F	p
	80-100	<.001	<.001	0.878	>.999	**0.977**	0.992	0.764	0.693	0.764	1 / 192	3.11E-20	>.999
	100-120	<.001	<.001	0.155	>.999	>.999	**>.999**	>.999	>.999	>.999			
	120-140	<.001	<.001	0.063	0.996	>.999	>.999	**>.999**	>.999	>.999	Intensity*Genotipe		
	140-160	<.001	<.001	0.008	0.869	>.999	>.999	>.999	**>.999**	>.999	df	F	p
	160-180	<.001	<.001	0.001	0.573	>.999	>.999	>.999	>.999	**>.999**	11 / 192	6.610	<.001

Fig S3K		Classification of DG Parvalbumin (PV)$^{+}$ interneurons according to the intensity of Aggrecan^{+} perineuronal nets (PNNs)											
		Two-way ANOVA									INTER-SUBJECT EFFECT		
		GSK-3-OE									Intensity		
		0-20	20-40	40-60	60-80	80-100	100-120	120-140	140-160	160-180	df	F	p
WT	0-20	**<.001**	0.934	0.004	<.001	<.001	<.001	<.001	<.001	<.001	11 / 192	42.360	<.001
	20-40	<.001	**0.002**	<.001	<.001	<.001	<.001	<.001	<.001	<.001			
	40-60	<.001	0.998	**0.026**	0.002	<.001	<.001	<.001	<.001	<.001	Genotipe		
	60-80	<.001	0.989	>.999	**>.999**	0.992	0.996	0.992	0.992	0.992	df	F	p
	80-100	<.001	0.217	>.999	>.999	**>.999**	>.999	>.999	>.999	>.999	1 / 192	5.483E-20	>.999
	100-120	<.001	0.117	>.999	>.999	>.999	**>.999**	>.999	>.999	>.999			
	120-140	<.001	0.139	>.999	>.999	>.999	>.999	**>.999**	>.999	>.999	Intensity*Genotipe		
	140-160	<.001	0.117	>.999	>.999	>.999	>.999	>.999	**>.999**	>.999	df	F	p
	160-180	<.001	0.117	>.999	>.999	>.999	>.999	>.999	>.999	**>.999**	11 / 192	12.500	<.001

Fig S3O	Frequency of mIPSCs in developmentally generated and 1-week-old adult-born DGCs of WT and GSK-3β-OE mice		INTER-SUBJECT EFFECT		
	Two-way ANOVA		Cell age		
			df	F	p
	GSK-3-OE		1 / 25	4.438	0.0453
WT	Embryonic	Adult	Genotipe		
Embryonic	0.5134	--	df	F	p
Adult	--	0.2494	1 / 25	3.731	0.0648
			Age*Genotipe		
			df	F	p
			1 / 25	0.05288	0.82

Fig S3P	Amplitude of mIPSCs in developmentally generated and 1-week-old adult-born DGCs of WT and GSK-3β-OE mice		INTER-SUBJECT EFFECT		
	Two-way ANOVA		Cell age		
			df	F	p
	GSK-3-OE		1 / 23	20.81	<.001
WT	Embryonic	Adult	Genotipe		
Embryonic	0.0946	--	df	F	p
Adult	--	>0.9999	1 / 23	3.739	0.0655
			Age*Genotipe		
			df	F	p
			1 / 23	0.88	0.3579

Fig S3Q	Slope of mIPSCs in developmentally generated and 1-week-old adult-born DGCs of WT and GSK-3β-OE mice		INTER-SUBJECT EFFECT		
	Two-way ANOVA		Cell age		
			df	F	p
	GSK-3-OE		1 / 23	20.08	<.001
WT	Embryonic	Adult	Genotipe		
Embryonic	0.0722	--	df	F	p
Adult	--	>0.9999	1 / 23	2.179	0.1535
			Age*Genotipe		
			df	F	p
			1 / 23	2.478	0.1291

SUPPLEMENTARY FIGURE S4:

Fig S4A	Sholl´s analysis of 1-week-old adult-born DGCs<br>REPEATED MEASURES ANOVA TEST<br>WT vs GSK-3-OE				
Distance to the soma (µm)	µm	p	INTER-SUBJECT EFFECT		
	0	>.999			
	10	>.999	Distance		
	20	0.062	df	F	p
	30	<.001	35 / 2160	88.070	<.001
	40	<.001			
	50	<.001	Genotipe		
	60	<.001	df	F	p
	70	>.999	1 / 2160	21.370	<.001
	80	>.999			
	90	>.999	Distance*Genotipe		
	100	>.999	df	F	p
	110	>.999	35 / 2160	3.068	<.001
	120	>.999			
	130	>.999			
	140	>.999			
	150	>.999			
	160	>.999			
	170	>.999			
	180	>.999			
	190	>.999			
	200	>.999			
	210	>.999			
	220	>.999			
	230	>.999			
	240	>.999			
	250	>.999			
	260	>.999			
	270	>.999			
	280	>.999			
	290	>.999			
	300	>.999			
	310	>.999			
	320	>.999			
	330	>.999			
	340	>.999			
	350	>.999			

Fig S4B	Total dendritic length of 1-week-old adult-born DGCs<br>Student t- Test		
	t	df	p
WT vs GSK-3-OE	2.736	58	0.008

Fig S4C	Sholl´s analysis of 2-week-old adult-born DGCs<br>REPEATED MEASURES ANOVA TEST<br>WT vs GSK-3-OE				
Distance to the soma (µm)	µm	p	INTER-SUBJECT EFFECT		
	0	>.999			
	10	>.999	Distance		
	20	0.005	df	F	p
	30	0.406	35 / 1044	110.400	<.001
	40	<.001			
	50	<.001	Genotipe		
	60	>.999	df	F	p
	70	0.176	1 / 1044	0.005	0.944
	80	<.001			
	90	<.001	Distance*Genotipe		
	100	0.27	df	F	p
	110	>.999	35 / 1044	3.804	<.001
	120	>.999			
	130	>.999			
	140	>.999			
	150	>.999			
	160	>.999			
	170	>.999			
	180	>.999			
	190	>.999			
	200	>.999			
	210	>.999			
	220	>.999			
	230	>.999			
	240	>.999			
	250	>.999			
	260	>.999			
	270	>.999			
	280	>.999			
	290	>.999			
	300	>.999			
	310	>.999			
	320	>.999			
	330	>.999			
	340	>.999			
	350	>.999			

Fig S4D	Total dendritic length of 2-week-old adult-born DGCs<br>Mann Whitney test	
	U	p
WT vs GSK-3-OE	422	0.686

Fig S4E	Sholl´s analysis of 3-week-old adult-born DGCs				
	REPEATED MEASURES ANOVA TEST				
	WT vs GSK-3-OE				
Distance to the soma (µm)	µm	p	INTER-SUBJECT EFFECT		
	0	>.999			
	10	>.999	Distance		
	20	>.999	df	F	p
	30	0.692	35 / 1584	77.120	<.001
	40	>.999			
	50	>.999	Genotipe		
	60	>.999	df	F	p
	70	>.999	1 / 1584	3.697	0.055
	80	>.999			
	90	>.999	Distance*Genotipe		
	100	>.999	df	F	p
	110	>.999	35 / 1584	2.031	<.001
	120	>.999			
	130	>.999			
	140	>.999			
	150	>.999			
	160	0.004			
	170	0.008			
	180	0.077			
	190	0.086			
	200	>.999			
	210	>.999			
	220	>.999			
	230	>.999			
	240	>.999			
	250	>.999			
	260	>.999			
	270	>.999			
	280	>.999			
	290	>.999			
	300	>.999			
	310	>.999			
	320	>.999			
	330	>.999			
	340	>.999			
	350	>.999			

Fig S4F	Total dendritic length of 3-week-old adult-born DGCs		
	Student t- Test		
	t	df	p
WT vs GSK-3-OE	0.2788	43	0.782

Fig S4G	Sholl´s analysis of 4-week-old adult-born DGCs<br>REPEATED MEASURES ANOVA TEST<br>WT vs GSK-3-OE				
Distance to the soma (µm)	µm	p	INTER-SUBJECT EFFECT		
	0	>.999			
	10	>.999	Distance		
	20	>.999	df	F	p
	30	>.999	35 / 1044	504.400	<.001
	40	>.999			
	50	>.999	Genotipe		
	60	>.999	df	F	p
	70	>.999	1 / 720	2.27E-14	>0.999
	80	>.999			
	90	>.999	Distance*Genotipe		
	100	>.999	df	F	p
	110	>.999	35 / 720	5.30E-15	>0.999
	120	>.999			
	130	>.999			
	140	>.999			
	150	>.999			
	160	>.999			
	170	>.999			
	180	>.999			
	190	>.999			
	200	>.999			
	210	>.999			
	220	>.999			
	230	>.999			
	240	>.999			
	250	>.999			
	260	>.999			
	270	>.999			
	280	>.999			
	290	>.999			
	300	>.999			
	310	>.999			
	320	>.999			
	330	>.999			
	340	>.999			
	350	>.999			

Fig S4H	Total dendritic length of 4-week-old adult-born DGCs<br>Student t- Test		
	t	df	p
WT vs GSK-3-OE	0.8483	51	0.4

Fig S4I	Sholl´s analysis of 8-week-old adult-born DGCs<br>REPEATED MEASURES ANOVA TEST<br>WT vs GSK-3-OE

Distance to the soma (µm)

µm	p
0	>.999
10	>.999
20	>.999
30	>.999
40	>.999
50	>.999
60	>.999
70	0.035
80	<.001
90	<.001
100	0.003
110	0.419
120	>.999
130	>.999
140	>.999
150	>.999
160	>.999
170	>.999
180	>.999
190	>.999
200	>.999
210	>.999
220	>.999
230	>.999
240	>.999
250	>.999
260	>.999
270	>.999
280	>.999
290	>.999
300	>.999
310	>.999
320	>.999
330	>.999
340	>.999
350	>.999

INTER-SUBJECT EFFECT		
**Distance**		
df	F	p
35 / 1044	74.680	<.001
**Genotipe**		
df	F	p
1 / 1044	30.940	<.001
**Distance*Genotipe**		
df	F	p
35 / 1044	2.005	<.001

Fig S4J	Total dendritic length of 8-week-old adult-born DGCs<br>Student t- Test		
	t	df	p
WT vs GSK-3-OE	5.068	57	<.001

SUPPLEMENTARY FIGURE S5:

Fig S5A	Density of PV$^+$ interneurons in the GCL		
	Student t- Test		
	t	df	p
WT vs GSK-3-OE	0.1908	15	0.851

Fig S5B	Density of PV$^+$ interneurons in the SGZ		
	Student t- Test		
	t	df	p
WT vs GSK-3-OE	1.335	16	0.201

Fig S5C	Density of PV$^+$ interneurons in the Hilus		
	Student t- Test		
	t	df	p
WT vs GSK-3-OE	1.562	16	0.138

Fig S5D	Lectin$^+$ fluorescence intensity in PNN of PV$^+$ interneurons located at the GCL		
	Student t- Test		
	t	df	p
WT vs GSK-3-OE	5.208	63	<.001

Fig S5E		Classification of PV$^+$ interneurons according to the intensity of Lectin$^+$ PNNs in the GCL											
		ANOVA									INTER-SUBJECT EFFECT		
		GSK-3-OE									Intensity		
		0-20	20-40	40-60	60-80	80-100	100-120	120-140	140-160	160-180	df	F	p
	0-20	**<.001**	<.001	0.272	>.999	>.999	>.999	>.999	>.999	>.999	11 / 192	17.560	<.001
	20-40	>.999	**>.999**	0.875	<.001	<.001	<.001	<.001	<.001	<.001			
	40-60	>.999	>.999	**0.946**	0.002	<.001	<.001	<.001	<.001	<.001	Genotipe		
	60-80	0.181	0.035	>.999	**0.989**	0.931	0.859	0.481	0.481	0.481	df	F	p
WT	80-100	0.052	0.007	>.999	>.999	**0.996**	0.985	0.807	0.807	0.807	1 / 192	2.95E-18	>.999
	100-120	<.001	<.001	0.272	>.999	>.999	**>.999**	>.999	>.999	>.999			
	120-140	<.001	<.001	0.272	>.999	>.999	>.999	**>.999**	>.999	>.999	Intensity*Genotipe		
	140-160	<.001	<.001	0.117	>.999	>.999	>.999	>.999	**>.999**	>.999	df	F	p
	160-180	<.001	<.001	0.06	>.999	>.999	>.999	>.999	>.999	**>.999**	11 / 192	3.604	<.001

Fig S5F	Lectin$^+$ fluorescence intensity in PNN of PV$^+$ interneurons located at the SGZ	
	Mann Whitney test	
	U	p
WT vs GSK-3-OE	475.5	<.001

Fig S5G		Classification of PV$^+$ interneurons according to the intensity of Lectin$^+$ PNNs in the SGZ											
		ANOVA									INTER-SUBJECT EFFECT		
		GSK-3-OE									Intensity		
		0-20	20-40	40-60	60-80	80-100	100-120	120-140	140-160	160-180	df	F	p
	0-20	**<.001**	<.001	0.038	0.982	>.999	>.999	>.999	>.999	>.999	11 / 192	22.310	<.001
	20-40	>.999	**>.999**	0.997	0.075	<.001	<.001	<.001	<.001	<.001			
	40-60	>.999	>.999	**0.99**	0.05	<.001	<.001	<.001	<.001	<.001	Genotipe		
	60-80	0.693	0.988	>.999	**0.992**	0.014	0.053	0.004	0.004	0.004	df	F	p
WT	80-100	<.001	0.013	0.553	>.999	**0.99**	>.999	0.92	0.92	0.92	1 / 192	9.67E-19	>.999
	100-120	<.001	<.001	0.026	0.965	>.999	**>.999**	>.999	>.999	>.999			
	120-140	<.001	<.001	0.005	0.774	>.999	>.999	**>.999**	>.999	>.999	Intensity*Genotipe		
	140-160	<.001	<.001	0.003	0.66	>.999	>.999	>.999	**>.999**	>.999	df	F	p
	160-180	<.001	<.001	0.001	0.486	>.999	>.999	>.999	>.999	**>.999**	11 / 192	4.020	<.001

Fig S5H	Lectin^{+} fluorescence intensity in PNN of PV^{+} interneurons located at the Hilus		
	Student t- Test		
	t	df	p
WT vs GSK-3-OE	2.066	50	0.044

Fig S5I		Classification of PV^{+} interneurons according to the intensity of Lectin^{+} PNNs in the Hilus											
		ANOVA									INTER-SUBJECT EFFECT		
		GSK-3-OE									Intensity		
		0-20	20-40	40-60	60-80	80-100	100-120	120-140	140-160	160-180	df	F	p
WT	0-20	**0.281**	0.981	0.292	0.7	0.068	0.095	0.119	0.036	0.119	11 / 192	14.200	<.001
	20-40	0.211	**0.96**	0.377	0.788	0.098	0.136	0.166	0.055	0.166			
	40-60	0.816	>.999	**0.039**	0.189	0.005	0.008	0.011	0.002	0.011	Genotipe		
	60-80	<.001	0.041	>.999	**>.999**	0.994	0.998	>.999	0.977	>.999	df	F	p
	80-100	<.001	<.001	>.999	>.999	**>.999**	>.999	>.999	>.999	>.999	1 / 192	2.20E-18	>.999
	100-120	<.001	<.001	>.999	>.999	>.999	**>.999**	>.999	>.999	>.999			
	120-140	<.001	0.002	>.999	>.999	>.999	>.999	**>.999**	>.999	>.999	Intensity*Genotipe		
	140-160	<.001	<.001	>.999	>.999	>.999	>.999	>.999	**>.999**	>.999	df	F	p
	160-180	<.001	<.001	>.999	>.999	>.999	>.999	>.999	>.999	**>.999**	11 / 192	2.571	0.005

Fig S5J	Aggrecan^{+} fluorescence intensity in PNN of PV^{+} interneurons located at the GCL		
	Student t- Test		
	t	df	p
WT vs GSK-3-OE	5.805	48	<.001

Fig S5K		Classification of PV^{+} interneurons according to the intensity of Aggrecan^{+} PNNs in the GCL											
		ANOVA									INTER-SUBJECT EFFECT		
		GSK-3-OE									Intensity		
		0-20	20-40	40-60	60-80	80-100	100-120	120-140	140-160	160-180	df	F	p
WT	0-20	**<.001**	>.999	0.598	0.154	0.096	0.096	0.096	0.096	0.096	11 / 192	24.110	<.001
	20-40	<.001	**0.409**	<.001	<.001	<.001	<.001	<.001	<.001	<.001			
	40-60	<.001	0.923	**0.011**	<.001	<.001	<.001	<.001	<.001	<.001	Genotipe		
	60-80	<.001	0.998	>.999	**>.999**	>.999	>.999	>.999	>.999	>.999	df	F	p
	80-100	<.001	0.491	>.999	>.999	**>.999**	>.999	>.999	>.999	>.999	1 / 192	2.10E-19	>.999
	100-120	<.001	0.347	>.999	>.999	>.999	**>.999**	>.999	>.999	>.999			
	120-140	<.001	0.347	>.999	>.999	>.999	>.999	**>.999**	>.999	>.999	Intensity*Genotipe		
	140-160	<.001	0.347	>.999	>.999	>.999	>.999	>.999	**>.999**	>.999	df	F	p
	160-180	<.001	0.347	>.999	>.999	>.999	>.999	>.999	>.999	**>.999**	11 / 192	8.874	0.031

Fig S5L	Aggrecan^{+} fluorescence intensity in PNN of PV^{+} interneurons located at the SGZ	
	Mann Whitney test	
	U	p
WT vs GSK-3-OE	182	<.001

Fig S5M		Classification of PV^{+} interneurons according to the intensity of Aggrecan^{+} PNNs in the SGZ											
		ANOVA									INTER-SUBJECT EFFECT		
		GSK-3-OE									Intensity		
		0-20	20-40	40-60	60-80	80-100	100-120	120-140	140-160	160-180	df	F	p
WT	0-20	**<.001**	>.999	0.3	0.14	0.044	0.044	0.044	0.044	0.044	11 / 192	26.750	<.001
	20-40	0.028	**<.001**	<.001	<.001	<.001	<.001	<.001	<.001	<.001			
	40-60	<.001	>.999	**0.461**	0.247	0.089	0.089	0.089	0.089	0.089	Genotipe		
	60-80	<.001	0.743	>.999	**>.999**	>.999	>.999	>.999	>.999	>.999	df	F	p
	80-100	<.001	0.379	>.999	>.999	**>.999**	>.999	>.999	>.999	>.999	1 / 192	8.27E-20	>.999
	100-120	<.001	0.272	>.999	>.999	>.999	**>.999**	>.999	>.999	>.999			
	120-140	<.001	0.272	>.999	>.999	>.999	>.999	**>.999**	>.999	>.999	Intensity*Genotipe		
	140-160	<.001	0.272	>.999	>.999	>.999	>.999	>.999	**>.999**	>.999	df	F	p
	160-180	<.001	0.272	>.999	>.999	>.999	>.999	>.999	>.999	**>.999**	11 / 192	8.704	<.001

Fig S5N	Aggrecan^{+} fluorescence intensity in PNN of PV^{+} interneurons located at the Hilus	
	Mann Whitney test	
	U	p
WT vs GSK-3-OE	130	0.003

Fig S5O		Classification of PV^{+} interneurons according to the intensity of Aggrecan^{+} PNNs in the Hilus											
		ANOVA									INTER-SUBJECT EFFECT		
		GSK-3-OE									Intensity		
		0-20	20-40	40-60	60-80	80-100	100-120	120-140	140-160	160-180	df	F	p
WT	0-20	**<.001**	<.001	<.001	<.001	<.001	<.001	<.001	<.001	<.001	11 / 192	47.470	<.001
	20-40	<.001	**0.997**	0.376	0.519	0.221	0.519	0.221	0.221	0.221			
	40-60	<.001	0.276	**0.004**	0.009	0.001	0.009	0.001	0.001	0.001	Genotipe		
	60-80	<.001	>.999	>.999	**>.999**	>.999	>.999	>.999	>.999	>.999	df	F	p
	80-100	<.001	>.999	>.999	>.999	**>.999**	>.999	>.999	>.999	>.999	1 / 192	1.67E-20	>.999
	100-120	<.001	0.996	>.999	>.999	>.999	**>.999**	>.999	>.999	>.999			
	120-140	<.001	0.996	>.999	>.999	>.999	>.999	**>.999**	>.999	>.999	Intensity*Genotipe		
	140-160	<.001	0.996	>.999	>.999	>.999	>.999	>.999	**>.999**	>.999	df	F	p
	160-180	<.001	0.996	>.999	>.999	>.999	>.999	>.999	>.999	**>.999**	11 / 192	5.497	<.001
